# Eukaryotic Microbial RNA Viruses—Acute or Persistent? Insights into Their Function in the Aquatic Ecosystem

**DOI:** 10.1264/jsme2.ME22034

**Published:** 2022-08-03

**Authors:** Syun-ichi Urayama, Yoshihiro Takaki, Yuto Chiba, Yanjie Zhao, Misa Kuroki, Daisuke Hagiwara, Takuro Nunoura

**Affiliations:** 1 Department of Life and Environmental Sciences, Laboratory of Fungal Interaction and Molecular Biology (donated by IFO), University of Tsukuba, 1–1–1 Tennodai, Tsukuba, Ibaraki 305–8577, Japan; 2 Microbiology Research Center for Sustainability (MiCS), University of Tsukuba, 1–1–1 Tennodai, Tsukuba, Ibaraki 305–8577, Japan; 3 Super-cutting-edge Grand and Advanced Research (SUGAR) Program, Japan Agency for Marine Science and Technology (JAMSTEC), 2–15 Natsushima-cho, Yokosuka, Kanagawa 237–0061, Japan; 4 Research Center for Bioscience and Nanoscience (CeBN), JAMSTEC, 2–15 Natsushima-cho, Yokosuka, Kanagawa 237–0061, Japan

**Keywords:** aquatic, RNA virus, eukaryote

## Abstract

Isolated RNA viruses mainly parasitize eukaryotes. RNA viruses either expand horizontally by infecting hosts (acute type) or coexist with the host and are vertically inherited (persistent type). The significance of persistent-type RNA viruses in environmental viromes (the main hosts are expected to be microbes) was only recently reported because they had previously been overlooked in virology. In this review, we summarize the host-virus relationships of eukaryotic microbial RNA viruses. *Picornavirales* and *Reoviridae* are recognized as representative acute-type virus families, and most of the microbial viruses in *Narnaviridae*, *Totiviridae*, and *Partitiviridae* are categorized as representative persistent-type viruses. Acute-type viruses have only been found in aquatic environments, while persistent-type viruses are present in various environments, including aquatic environments. Moreover, persistent-type viruses are potentially widely spread in the RNA viral sequence space. This emerging evidence provides novel insights into RNA viral diversity, host-virus relationships, and their history of co-evolution.

An RNA virus has single- or double-stranded RNA as its genome. The genome sizes of RNA viruses range from several kb to several tens of kb, and some harbor segmented genomes depending on the virus group/species (King *et al.*, 2012; Koonin *et al.*, 2020). To date, most RNA viruses have been isolated from eukaryotes, and more than 50% of isolated viruses from eukaryotes are RNA viruses (Nasir *et al.*, 2014). Since the majority of eukaryotes are expected‍ ‍to be infected by RNA viruses, RNA viruses and eukaryotes likely have a long history of co-evolution (Koonin *et al.*, 2015). In contrast, no or very few RNA virus families‍ ‍have been isolated from archaea and bacteria, respectively; however, the diversity of prokaryotic RNA viruses was‍ ‍very recently expanded by metatranscriptomics and subsequent *in silico* ana­lyses (Callanan *et al.*, 2020; Neri, U., *et al.* 2022. A five-fold expansion of the global RNA virome reveals multiple new clades of RNA bacteriophages. bioRxiv https://doi.org/10.1101/2022.02.15.480533).

Historically, RNA viruses have been detected as infectious causative agents in humans and economically important plants and animals (Beijerinck, 1898; Loeffler and Frosch, 1898; Reed and Carroll, 1901). However, in the last several decades, fungal RNA viruses that persistently infect their hosts without causing clear phenotypic changes have been discovered, although a few have been found in edible mushrooms and pathogenic fungi that cause phenotypic symptoms (Nuss, 2005; Roossinck, 2014). Moreover, similar “cryptic” RNA viruses are widely present in plants (Roossinck *et al.*, 2010). To obtain a more detailed understanding of RNA viruses, Roossinck categorized the lifestyles of plant/fungal viruses into two types, persistent and acute (Roossinck, 2010, 2012, 2015). Briefly, viruses with a persistent lifestyle are transmitted vertically via cell division, while viruses with an acute lifestyle are most frequently transmitted horizontally (Fig. 1). This classification is completely different from “acute infection”, “persistent (latent) infection”, and “chronic infection” in human infections. These clinical categories indicate the course of viral dose/activity or the state of disease in humans. For example, hepatitis C virus (HCV), a positive-sense single-stranded RNA virus, sometimes establishes a chronic infection after an acute infection. However, this does not represent a change in the viral lifestyle. HCV is categorized as an acute-type RNA virus by Roossinck’s criteria because it mainly spreads by horizontal transmission.

The acute and persistent types have both been identified in eukaryotic microbial lineages of fungi and protists. Furthermore, viral metagenomics enables us to identify diverse RNA viruses and infer their lifestyles. To date, a large diversity of RNA viruses has been revealed in soil and aquatic microbial ecosystems (Culley *et al.*, 2006; Suttle, 2016; Urayama *et al.*, 2018a; Starr *et al.*, 2019; Wolf *et al.*, 2020; Edgar *et al.*, 2022; Zayed *et al.*, 2022), in addition to biological samples from organisms, such as invertebrates, deep-sea animals, and microbial consortia associated with sponges, macroalgae, and lichens (Shi *et al.*, 2016; Urayama *et al.*, 2018b, 2020a, 2020b; Chiba *et al.*, 2020). In this review, we summarize eukaryotic microbial RNA viruses based on their lifestyles and discuss their significance in microbial ecosystems. This review focuses on RNA viruses excluding retroviruses. Retroviruses are distinct from other RNA viruses in their life cycle because they are embedded into the host genome, whereas other RNA viruses are not. Retroviruses have a mixed strategy of vertical and horizontal transmission and change their strategy depending on the life stage.

## Microbial RNA viruses have two life cycle strategies

**Fig. 1.**
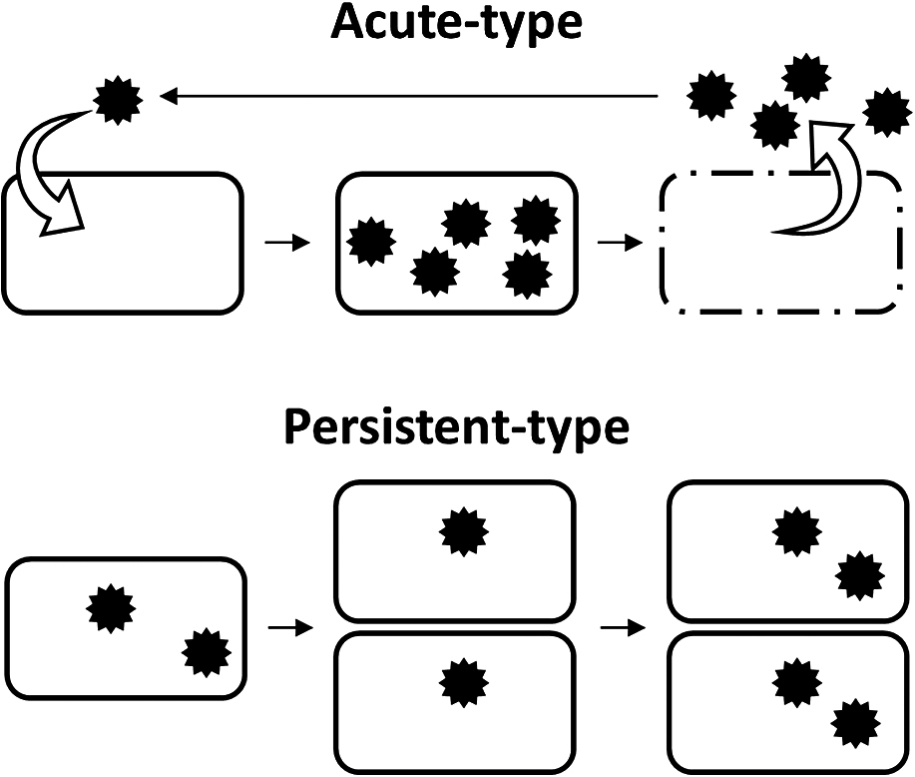
Schematic of transmission routes of microbial RNA viruses.

We define acute- and persistent-type life cycles in microbial RNA viruses based on Roossinck’s definition (Roossinck, 2010) (Fig. 1). Acute-type viruses are mainly transmitted horizontally via extracellular viral particles. These viruses have the ability to enter host cells from the outside and exit host cells to the outside. During the viral life cycle, acute-type viruses often cause “disease” as represented by cell lysis (Tomaru *et al.*, 2004). In contrast, persistent-type viruses are mainly transmitted vertically via the cell division of their host without cell lysis (Nuss, 2005). These viruses remain associated with their hosts for many generations with nearly 100% vertical transmission, and sometimes lack the ability to enter and exit host cells. In addition, some persistent-type RNA viruses lack capsid proteins (Dolja and Koonin, 2012). No gene is shared only in persistent-type RNA viruses. Persistence and vertical transmission in viruses has been suggested to correlate with commensal or mutualistic lifestyles (Roossinck, 2011; Marquez and Roossinck, 2012).

The host cell phenotype is an attractive subject in virology, and persistent- and acute-type lifestyles overlap with symbiosis and antagonism, respectively. However, difficulties are associated with the classification of viruses according to the phenotype of the host organism because the host-virus relationship changes depending on the surrounding conditions (Xu *et al.*, 2008). Furthermore, we cannot test all environmental conditions that may affect the host phenotype. Therefore, we defined the two types of viruses based on the viral transmission route in this review.

Heterosigma akashiwo RNA virus (HaRNAV) (Tai *et al.*, 2003; Lang *et al.*, 2004) is a well-known acute-type RNA virus associated with microbial eukaryotes in aquatic ecosystems. HaRNAV was isolated from a *Heterosigma akashiwo* culture that showed the lysis of host cells after the inoculation of a virus particle fraction from seawater. To maintain HaRNAV in lab conditions, a 0.22-μm filtered cell lysate including HaRNAV is inoculated into *H. akashiwo* cultures, and after cell lysis, the lysate is used as an inoculum. In this case, since the host is re-infected with the daughter virus obtained by filter filtration from the lytic solution, we defined the RNA virus as an acute-type virus. Based on the genome sequence of HaRNAV, it belongs to *Marnaviridae* (Lang *et al.*, 2021), which includes several acute-type microbial RNA viral genera, such as *Marnavirus* and *Labyrnavirus* (Lang *et al.*, 2004; Takao *et al.*, 2006).

Among persistent-type RNA viruses, Saccharomyces cerevisiae virus L-A (ScV-L-A) (Wickner, 1996) is a representative strain. The presence of RNA viruses may be confirmed by long cellular dsRNA because it only consists of dsRNA viral genomes and/or replicative intermediates of non-retro ssRNA viruses (Morris and Dodds, 1979). ScV-‍L-‍A does not confer a detectable phenotype upon its host (yeast) cells (Schmitt and Breinig, 2002), and viral dsRNA is maintained in the host cell and transmitted to daughter cells via cell division. ScV-L-A may be maintained and amplified in *S. cerevisiae* cells under laboratory conditions. In this case, since the RNA virus is maintained by a host sub-culture, we define the RNA virus as a persistent-type virus. ScV-‍L-‍A belongs to the family *Totiviridae*, which includes other known totiviruses isolated from protists (Goodman *et al.*, 2011).

The definitions of acute- and persistent-type viruses are not applicable in some exceptional cases. For example, fungal persistent-type RNA viruses are mainly transmitted to daughter cells, but may also be transmitted between closely related fungal strains through anastomosis (Liu *et al.*, 2003; Vainio *et al.*, 2011). On a geological time scale, the transmission of RNA viruses between plants and fungi may also have occurred (Nibert *et al.*, 2014; Roossinck, 2019; Bian *et al.*, 2020).

## Isolated microbial RNA viruses

We herein summarized and grouped isolated microbial RNA viruses based on the infection type (Table S1). Microbial RNA viruses with acute-type lifestyles have been obtained from aquatic host strains (Sadeghi *et al.*, 2021). Among them, ssRNA viruses that lyse host cells have been‍ ‍reported, such as Heterosigma akashiwo RNA virus (HaRNAV) (*Picornavirales*), which infects the toxic bloom-forming raphidophyte *Heterosigma akashiwo* (Raphidophyceae) (Tai *et al.*, 2003); Heterocapsa circularisquama RNA virus 01 (*Alvernaviridae*), which infects the bivalve-killing dinoflagellate *Heterocapsa circularisquama* (Dinophyceae) (Tomaru *et al.*, 2004); and Aurantiochytrium single-stranded RNA virus 01 (*Picornavirales*), which infects the marine fungoide *Schizochytrium* sp. (Thraustochytriaceae) (Takao *et al.*, 2005). The dsRNA virus, Micromonas pusilla reovirus (*Reoviridae*), which infects and lyses the marine photosynthetic protist *Micromonas pusilla* (Mamiellophyceae) (Brussaard *et al.*, 2004), has also been isolated.

In contrast, microbial RNA viruses with persistent-type lifestyles have mainly been identified using intracellular dsRNA (a mole­cular biomarker) or RNA-seq technology. This type of RNA virus has been exclusively studied and identified in fungi (Ghabrial *et al.*, 2015; Kotta-Loizou and Coutts, 2017). To date, identified dsRNA viruses include unclassified RNA viruses that infect the cultivated mushroom *Agaricus bisporus* (van der Lende *et al.*, 1994); Penicillium chrysogenum virus (*Chrysoviridae*), which infects the penicillin-producing strains of *Penicillium chrysogenum* (Banks *et al.*, 1969); and Saccharomyces cerevisiae virus L-A (*Totiviridae*), which infects the budding yeast *S. cerevisiae* (Wickner, 1996). Oomycetes (Oomycota) have also been subject to dsRNA-based surveillance, and the plant pathogens *Phytophthora infestans* and *Asparagus officinalis* are known to harbor Phytophthora infestans RNA virus 3 (unclassified) and Phytophthora endornavirus 2 (and 3) (*Endornaviridae*), respectively (Cai *et al.*, 2013; Uchida *et al.*, 2021). In addition, protozoans, such as *Trichomonas*, *Leishmania*, and *Giardia*, harbor persistent-type RNA viruses (Wang and Wang, 1986; Stuart *et al.*, 1992; Khoshnan and Alderete, 1994); however, the presence of extracellular infection routes has been suggested (Wang and Wang, 1986; Torrecilhas *et al.*, 2020).

RNA viruses with acute-type lifestyles have been detected in aquatic environments, while those with persistent-type lifestyles have been found in terrestrial environments. However, ssRNA and dsRNA viruses with persistent-type lifestyles have both been recently identified from a marine oomycete strain in the genus *Halophytophthora* (Botella *et al.*, 2020) and a marine fungal strain isolated from the seagrass *Posidonia oceanica* (Nerva *et al.*, 2016). The identification of these strains was based on high-throughput sequencing methods, while culture-dependent isolation has typically been used to identify viruses in aquatic research. However, in terrestrial environments, few attempts have been made to obtain acute-type RNA viruses. We cannot exclude the possibility that the different distribution pattern observed between the two lifestyle types is a result of methodological bias in virus detection and isolation; however, acute-type RNA viruses may have advantages in dispersal via viral particles to access new host cells in aquatic environments. In the case of DNA phages, a number of theories have been proposed, including Kill-the-Winner and Piggyback-the-Winner (Obeng *et al.*, 2016; Pratama and van Elsas, 2018). Theoretical modeling in addition to further studies will provide more detailed insights into the distribution of acute- and persistent-type RNA viruses under diverse environmental conditions.

## Persistent and acute RNA viruses in the RNA viral sequence space

**Fig. 2.**
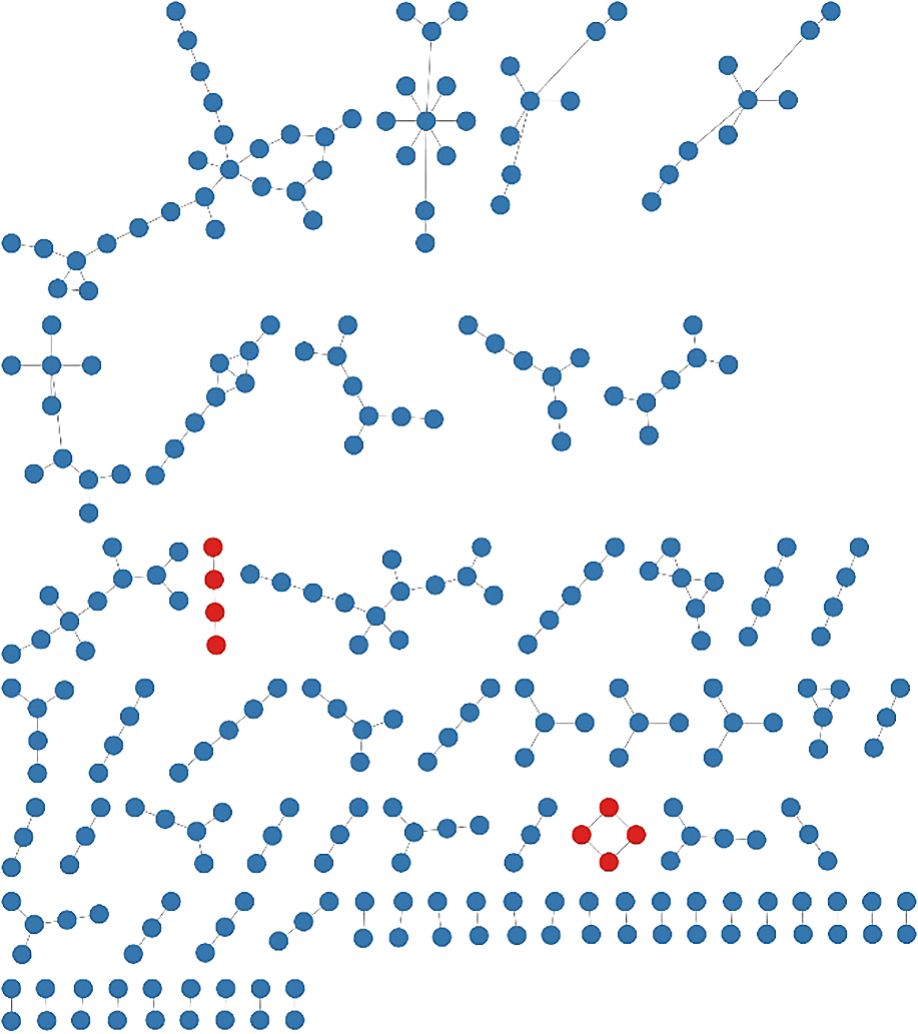
A total of 293 isolated acute- and persistent-type microbial RNA viruses in sequence-based clusters. Colored circles indicate the lifestyle of each RNA virus: red, acute; blue, persistent. In total, 315 isolated microbial eukaryotic RNA viruses were collected from the manually curated Virus-Host database (Mihara *et al.*, 2016) downloaded on 2021.12.09. Sequences were clustered at 70% amino acid identity. Representative sequences were applied to a network ana­lysis with MOCASSIN-prot (Keel *et al.*, 2018).

**Fig. 3.**
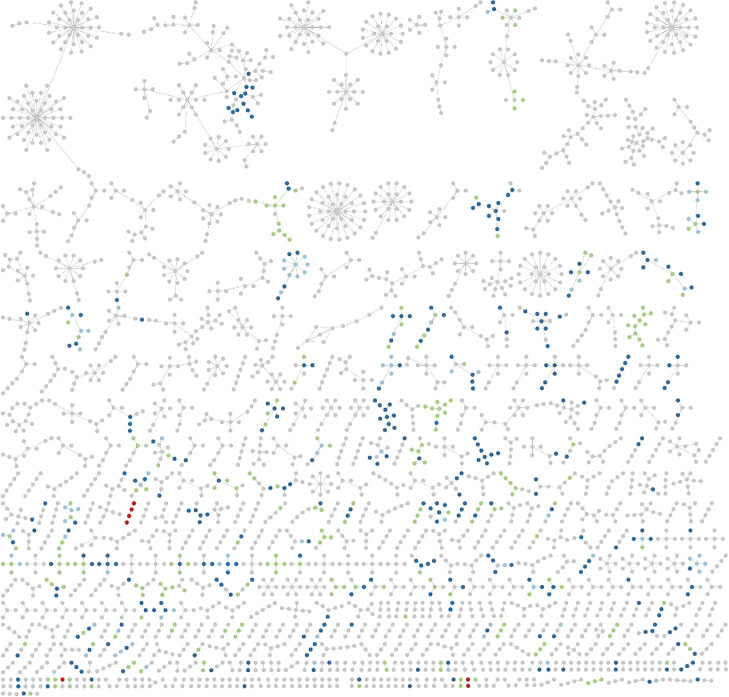
RdRp sequences of known RNA viruses obtained from the Identical Protein Groups resource (https://www.ncbi.nlm.nih.gov/ipg) with keywords “rna dependent rna polymerase” and “viruses”. After the removal of short (<200 aa) sequences, RdRp sequences were clustered at 70% using CD-HIT (Huang *et al.*, 2010). Representative sequences were applied to a network ana­lysis with MOCASSIN-prot (Keel *et al.*, 2018). Colored circles indicate percent identity to persistent- or acute-type microbial RNA viruses: blue 100% to persistent; sky blue >70% to persistent; green >50% to persistent; red 100% to acute (>70 and 50% to acute were not identified).

To visualize the richness of persistent-type RNA viruses in the sequence space, RNA viruses isolated from eukaryotic microorganisms were classified into acute- or persistent-types based on culture-dependent laboratory observations (Table S1), as described above. In total, 96% (304/314) of eukaryotic microbial RNA viruses were recognized as persistent types (Fig. 2), while we were unable to exclude the possibility of experimental biases in culture-dependent ana­lyses to obtain acute-type RNA viruses in eukaryotic microorganisms. To predict the distribution of persistent-type RNA viruses in the total RNA virome sequence space, we used this list as an operational reference virus list because of the lack of knowledge on persistent-type RNA viruses in other host organisms and limited metagenomic data. Based on the operational reference virus list, RNA viral clusters including all known viral RdRp sequences were analyzed, and clusters including RNA viruses related to microbial persistent-type viruses (>50% amino acid similarity) were distinguished from those of acute-type viruses (Fig. 3). In our current ana­lysis, more than 1/3 of the clusters (including five or more sequences) were predicted to include persistent-type viruses. Since the number of isolated persistent-type RNA viruses is limited, the true richness of persistent-type RNA viruses needs to be higher than that estimated.

## Microbial RNA viruses in metagenomic data

Metagenomics is a powerful tool for identifying DNA viromes in nature (Breitbart *et al.*, 2002; Edwards and Rohwer, 2005; Suttle, 2016), and its use for RNA viromes has markedly increased (Culley *et al.*, 2006, 2010; Steward *et al.*, 2013; Wolf *et al.*, 2020; Edgar *et al.*, 2022; Zayed *et al.*, 2022). RNA virome research is conducted as either metagenomics (metatranscriptomics) of RNA virus particles or as cellular transcriptomics. RNA virus particles in extracellular environments are expected to predominantly be acute-type viruses, and cellular RNA may harbor viral RNA from both acute- and persistent-type viruses (Fig. 1). Accordingly, cellular RNA-specific viruses are expected to be of the persistent type. In addition, dsRNA-seq for cellular RNA has also been used to detect cellular viral RNA sequences in some studies in order to more efficiently retrieve RNA viral sequences (Decker and Parker, 2014; Decker *et al.*, 2019; Izumi *et al.*, 2021).

In aquatic environments, most of the acute-type RNA viruses identified from extracellular viral particles belong to specific RNA viral lineages, such as *Picornavirales* (ssRNA) and *Reoviridae* (dsRNA) (Culley *et al.*, 2006, 2014; Djikeng *et al.*, 2009; Steward *et al.*, 2013; Culley, 2017; Urayama *et al.*, 2018a; Wolf *et al.*, 2020). In addition, *Picobirnaviridae* (dsRNA) viruses that may infect bacterial hosts were identified as a dominant RNA virus population (Ghosh and Malik, 2021; Neri, U., *et al.* 2022. bioRxiv https://doi.org/10.1101/2022.02.15.480533.). The contribution of *Picornavirales* to viral lysis via their acute lifestyle and subsequent release of organic matter (previously defined as the virus shunt in DNA virus studies [Wilhelm and Suttle, 1999; Zimmerman *et al.*, 2020]) was suggested based on the correlation between the relative abundance of transcripts related to *Picornavirales* and the amount of particulate organic carbon in pelagic ecosystems (Kaneko *et al.*, 2021). These findings are also consistent with the results of culture-dependent isolation experiments as described above.

Based on pelagic microbial cellular RNA, in addition to *Picornavirales*, *Reoviridae*, and *Picobirnaviridae*, a wide range of RNA virus groups has been identified (Urayama *et al.*, 2016, 2018a). Among them, the dominant members belong to *Narnaviridae* (ssRNA) and *Partitiviridae* (dsRNA), which have been recognized as persistent-type RNA viruses associated with fungi and plants/eukaryotic microorganisms, respectively (Hillman and Cai, 2013; Nibert *et al.*, 2014). Moreover, RNA virome studies have been conducted on macrocolonies of eukaryotic microorganisms, such as *Delisea pulchra* (red algae) and *Scytosiphon lomentaria* (brown algae). An association with *Totiviridae* (dsRNA) viral genomes, which are a persistent type, is generally observed; however, diverse RNA virus groups constitute the viromes (Lachnit *et al.*, 2016; Urayama *et al.*, 2016; Chiba *et al.*, 2020). In contrast, *Leviviridae* (ssRNA bacteriophage) and *Narnaviridae* virus operational taxonomic units (OTUs) have been identified as the predominant populations in soil RNA viromes, although the physical separation of cellular and viral particles has not been examined (Starr *et al.*, 2019).

**Fig. 4.**
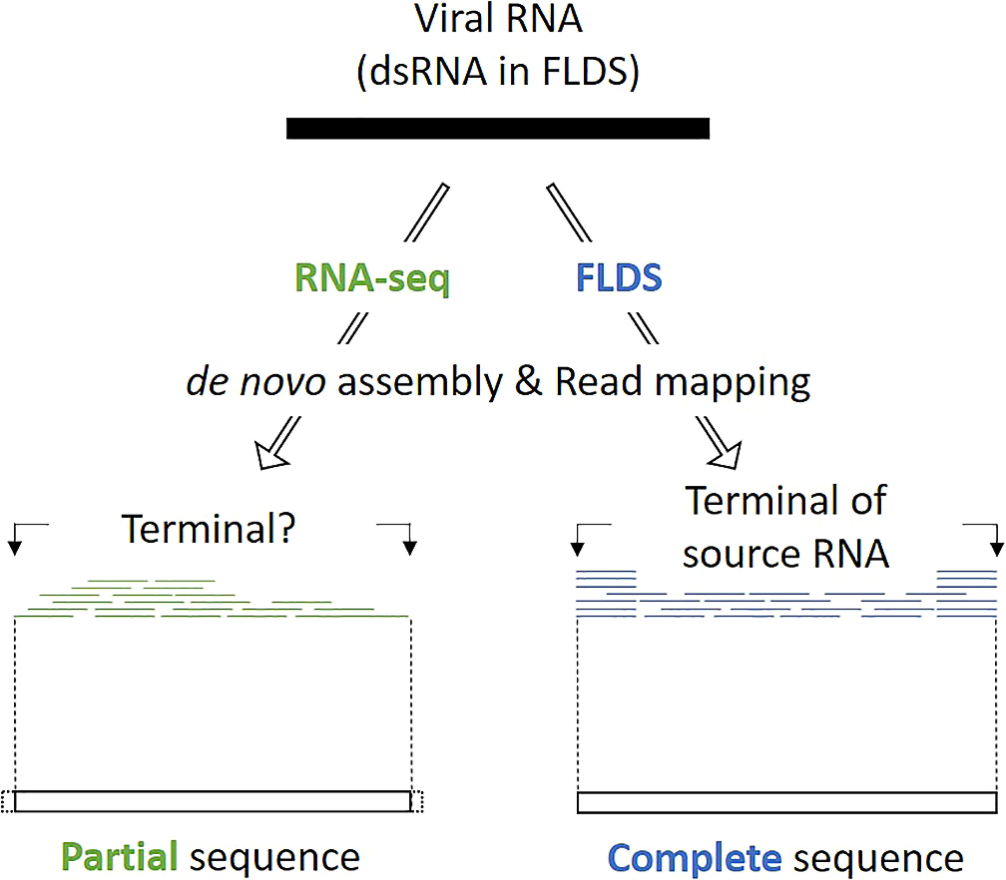
Differences in conventional RNA-seq and FLDS for resultant contigs. In a *de novo* ana­lysis, terminal sequence positions were not defined by RNA-seq data. However, FLDS data enabled us to identify terminal sequence positions because FLDS sequence reads included RACE (Rapid Amplification of cDNA Ends), which is widely used to assess the terminal sequences of RNA molecules (Urayama *et al.*, 2016).

**Fig. 5.**
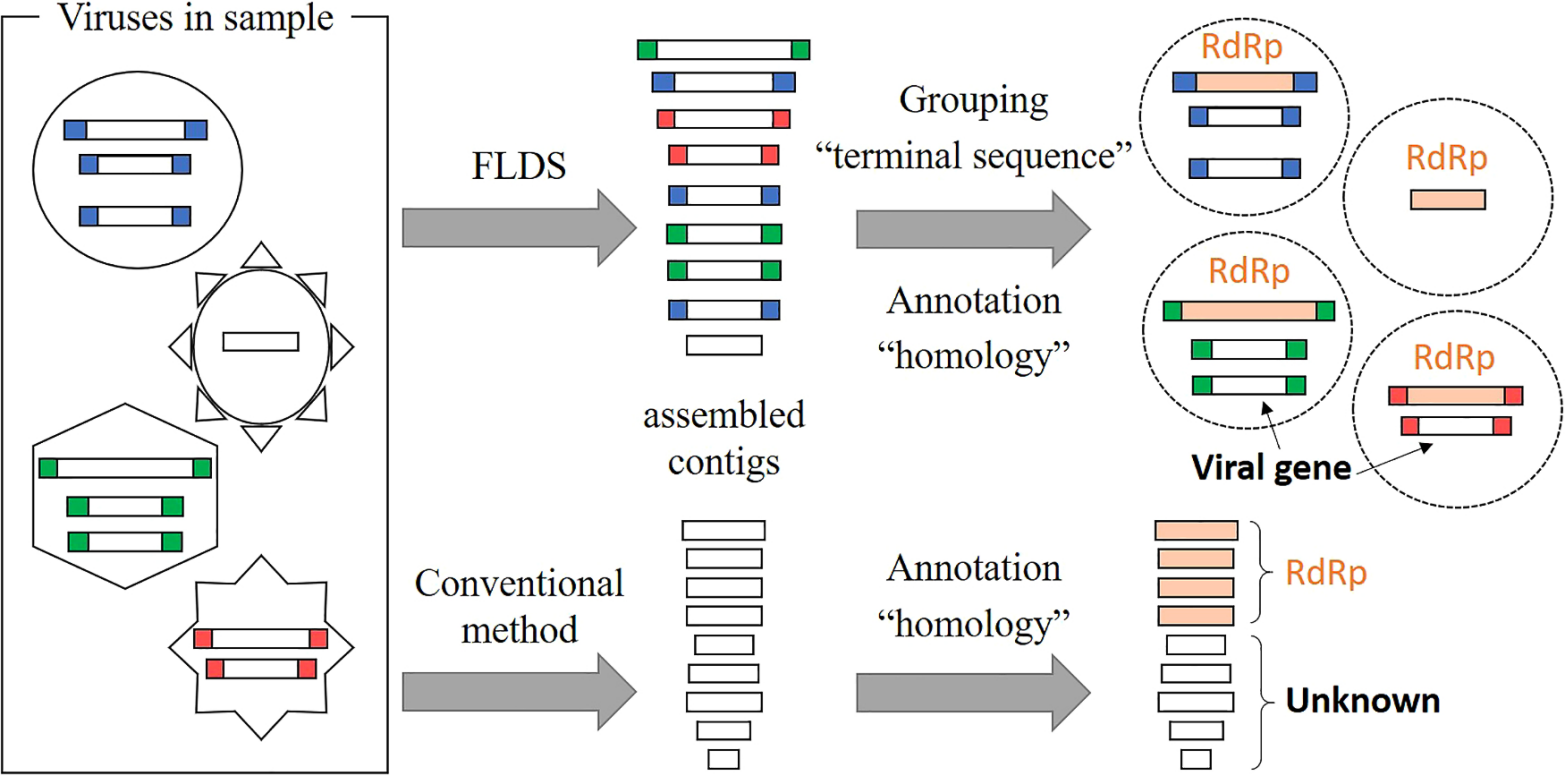
Concept of RNA viral genome reconstruction based on conserved terminal sequences in segmented RNA virus genomes. Many segmented RNA viruses have conserved 5′- and 3′-terminal sequences (colored boxes). FLDS enabled us to obtain full-length RNA sequences, which are difficult to obtain with conventional RNA-seq technologies (Fig. 2). Based on terminal sequences, we reconstructed RNA viral genomes. If RdRp is identified in a potential RNA viral genome, we predict that other RNA sequences, which do not show significant similarity to known RNA viruses, will be segments of the RNA virus.

We previously compared viral dsRNA in extracellular virus particle fractions and cellular dsRNA from surface seawater (Urayama *et al.*, 2018a). We showed 3.7- to 14.9-fold more dsRNA viral OTUs in cellular dsRNA metatranscriptomes than in extracellular dsRNA metatranscriptomes. Although we cannot rule out the possibility that some dsRNA virus particles were lost during sample processing, cellular dsRNA viruses, presumed to be of the persistent type, were abundant in the aquatic environment. In the present and related studies, we used a combination of fragmented and primer-ligated dsRNA sequencing (FLDS) and genome reconstruction *in silico* to identify viral sequences that are distinct from those in public databases. FLDS is a sequencing method that is applicable to long dsRNAs and enables the retrieval of the complete genomic sequences of both dsRNA and ssRNA viruses (Urayama *et al.*, 2016; Fukasawa *et al.*, 2020; Hirai *et al.*, 2021; Uehara-Ichiki *et al.*, 2021). In this technique, the terminal sequences of an RNA virus genome or genome segment may both be identified by sequence read mapping (Fig. 4). Therefore, it may be used to reconstruct multi-segmented RNA viral genomes based on terminally conserved sequences among segments (Fig. 5). In other words, a group of contigs sharing either/both terminal end sequences may represent a multi-segmented RNA virus genome. Moreover, if an RdRp gene or other virus proteins in any one of the segments in a reconstructed genome is identified based on sequence-dependent methods, all of the segments in the reconstructed genome may subsequently be identified as viral genome segments. We previously identified approximately 800 novel RNA viral genes that did not show significant (e-value less than 1×10^–5^) similarities to known protein sequences (Urayama *et al.*, 2018a). Therefore, FLDS provides opportunities for deep surveys of RdRp and the identification of novel genes that are distinct from the viral genes in public databases.

## Function of microbial RNA viruses in aquatic ecosystems

Knowledge on the ecological functions of acute-type microbial RNA viruses is based on the effects of host cell lysis. For example, previous studies suggested the contribution of RNA viruses to the virus shunt in pelagic zones (Kaneko *et al.*, 2021) and soil environments (Starr *et al.*, 2019). In addition, a sequence match between a CRISPR spacer and a bacterial RNA virus was reported (Wolf *et al.*, 2020). In contrast, their impacts on the evolution of microbial hosts have not yet been revealed. In the case of animals, RNA viral sequences incorporated into the host genome protect the host organism from related RNA viral infections (Suzuki *et al.*, 2020). However, a similar system has not been reported for microbes infected with RNA viruses. The ability to mediate horizontal gene transfer, which is often reported for DNA viruses, has also not been reported.

In contrast, persistent-type microbial RNA viruses have a wide range of effects through the manipulation of their host organisms. For example, fungi with an RNA virus may induce thermal tolerance to plants, while those without the RNA virus do not (Marquez *et al.*, 2007). Persistent-type RNA viruses also influence biological interactions between hosts and other organisms by changing secondary metabolites and pathogenicity (Chiba *et al.*, 2009; Zhang *et al.*, 2014; Aihara *et al.*, 2018; Okada *et al.*, 2018; Takahashi-Nakaguchi *et al.*, 2019; Ninomiya *et al.*, 2020). In some cases, persistent-type RNA viruses provide advantages to their hosts under specific conditions (Okada *et al.*, 2018). Therefore, these viruses may affect the adaptation of microbes to environmental challenges (Bao and Roossinck, 2013). Many of these are examples in fungi, which serve as model systems for persistent-type RNA viral research; however, their functions in aquatic environments remain largely unknown. Nevertheless, the host-virus relationship and the impact of persistent-type RNA viral infection on the physiology of their hosts in terrestrial ecosystems imply that persistent-type RNA viruses in aquatic ecosystems also influence host physiology and subsequent ecological functions, including the niche adaptation of their host organisms.

Climate change due to global warming and ocean acidification, is an important issue for our planet. These environmental changes are expected to affect pelagic microbial ecosystems, and other impacts on marine microbial ecosystems and subsequent biogeochemical cycles may also accelerate environmental changes. Therefore, observations of the marine microbial community are essential for understanding microbial responses to a changing ocean. Furthermore, current updates in our understanding of the RNA virome suggest that acute- and persistent-type microbial RNA viruses both play a significant role in biogeochemical cycles via the viral shunt and the regulation of host physiology. Accordingly, we need to pay close attention to marine RNA viromes in the changing ocean even though pelagic RNA viromes have so far been overlooked in marine microbial ecology.

## Citation

Urayama, S., Takaki, Y., Chiba, Y., Zhao, Y., Kuroki, M., Hagiwara, D., and Nunoura, T. (2022) Eukaryotic Microbial RNA Viruses—Acute or Persistent? Insights into Their Function in the Aquatic Ecosystem. *Microbes Environ ***37**: ME22034.

https://doi.org/10.1264/jsme2.ME22034

## Supplementary Material

Supplementary Material

## References

[B1] Aihara, M., Urayama, S., Le, M.T., Katoh, Y., Higashiura, T., Fukuhara, T., et al. (2018) Infection by Magnaporthe oryzae chrysovirus 1 strain A triggers reduced virulence and pathogenic race conversion of its host fungus, Magnaporthe oryzae. J Gen Plant Pathol 84: 92–103.

[B2] Banks, G.T., Buck, K.W., Chain, E.B., Darbyshire, J.E., and Himmelweit, F. (1969) Virus-like particles in penicillin producing strains of Penicillium chrysogenum. Nature 222: 89–90. 10.1038/222089b0 5191625

[B3] Bao, X., and Roossinck, M.J. (2013) Multiplexed interactions: viruses of endophytic fungi. In *Advances in Virus Research*. Amsterdam: Elsevier, pp. 37–58. 10.1016/B978-0-12-394315-6.00002-7 23498902

[B4] Beijerinck, M.W. (1898) Concerning a contagium vivum fluidum as cause of the spot disease of tobacco leaves. Phytopathol Classics 7: 33–52.

[B5] Bian, R., Andika, I.B., Pang, T., Lian, Z., Wei, S., Niu, E., et al. (2020) Facilitative and synergistic interactions between fungal and plant viruses. Proc Natl Acad Sci U S A 117: 3779–3788. 32015104 10.1073/pnas.1915996117 PMC7035501

[B6] Botella, L., Janoušek, J., Maia, C., Jung, M.H., Raco, M., and Jung, T. (2020) Marine oomycetes of the genus Halophytophthora harbor viruses related to bunyaviruses. Front Microbiol 11: 1467. 32760358 10.3389/fmicb.2020.01467 PMC7375090

[B7] Breitbart, M., Salamon, P., Andresen, B., Mahaffy, J.M., Segall, A.M., Mead, D., et al. (2002) Genomic ana­lysis of uncultured marine viral communities. Proc Natl Acad Sci U S A 99: 14250–14255. 12384570 10.1073/pnas.202488399 PMC137870

[B8] Brussaard, C.P., Noordeloos, A.A., Sandaa, R.A., Heldal, M., and Bratbak, G. (2004) Discovery of a dsRNA virus infecting the marine photosynthetic protist Micromonas pusilla. Virology 319: 280–291. 14980488 10.1016/j.virol.2003.10.033

[B9] Cai, G., Krychiw, J.F., Myers, K., Fry, W.E., and Hillman, B.I. (2013) A new virus from the plant pathogenic oomycete Phytophthora infestans with an 8‍ ‍kb dsRNA genome: the sixth member of a proposed new virus genus. Virology 435: 341–349. 23146209 10.1016/j.virol.2012.10.012

[B10] Callanan, J., Stockdale, S.R., Shkoporov, A., Draper, L.A., Ross, R.P., and Hill, C. (2020) Expansion of known ssRNA phage genomes: from tens to over a thousand. Sci Adv 6: eaay5981. 32083183 10.1126/sciadv.aay5981 PMC7007245

[B11] Chiba, S., Salaipeth, L., Lin, Y.H., Sasaki, A., Kanematsu, S., and Suzuki, N. (2009) A novel bipartite double-stranded RNA Mycovirus from the white root rot Fungus Rosellinia necatrix: mole­cular and biological characterization, taxonomic considerations, and potential for biological control. J Virol 83: 12801–12812. 19828620 10.1128/JVI.01830-09 PMC2786845

[B12] Chiba, Y., Tomaru, Y., Shimabukuro, H., Kimura, K., Hirai, M., Takaki, Y., et al. (2020) Viral RNA genomes identified from marine macroalgae and a diatom. Microbes Environ 35: ME20016. 32554943 10.1264/jsme2.ME20016 PMC7511793

[B13] Culley, A. (2017) New insight into the RNA aquatic virosphere via viromics. Virus Res 244: 84–89. 29138044 10.1016/j.virusres.2017.11.008

[B14] Culley, A.I., Lang, A.S., and Suttle, C.A. (2006) Metagenomic ana­lysis of coastal RNA virus communities. Science 312: 1795–1798. 16794078 10.1126/science.1127404

[B15] Culley, A.I., Suttle, C.A., and Steward, G.F. (2010) Characterization of the diversity of marine RNA viruses. In *Manual of Aquatic Viral Ecology*. Wilheim, S., Weinbauer, M., and Suttle, C. (eds). Waco, TX: American Society of Limnology and Oceanography (ASLO), pp. 193–201.

[B16] Culley, A.I., Mueller, J.A., Belcaid, M., Wood-Charlson, E.M., Poisson, G., and Steward, G.F. (2014) The characterization of RNA viruses in tropical seawater using targeted PCR and metagenomics. mBio 5: e01210–01214. 24939887 10.1128/mBio.01210-14 PMC4068258

[B17] Decker, C.J., and Parker, R. (2014) Analysis of double-stranded RNA from microbial communities identifies double-stranded RNA virus-like elements. Cell Rep 7: 898–906. 24767992 10.1016/j.celrep.2014.03.049 PMC4117469

[B18] Decker, C.J., Steiner, H.R., Hoon-Hanks, L.L., Morrison, J.H., Haist, K.C., Stabell, A.C., et al. (2019) dsRNA-Seq: identification of viral infection by purifying and sequencing dsRNA. Viruses 11: 943. 31615058 10.3390/v11100943 PMC6832592

[B19] Djikeng, A., Kuzmickas, R., Anderson, N.G., and Spiro, D.J. (2009) Metagenomic ana­lysis of RNA viruses in a fresh water lake. PLoS One 4: e7264. 19787045 10.1371/journal.pone.0007264 PMC2746286

[B20] Dolja, V.V., and Koonin, E.V. (2012) Capsid-less RNA viruses. eLS. URL https://doi.org/10.1002/9780470015902.a0023269

[B21] Edgar, R.C., Taylor, J., Lin, V., Altman, T., Barbera, P., Meleshko, D., et al. (2022) Petabase-scale sequence alignment catalyses viral discovery. Nature 602: 142–147. 35082445 10.1038/s41586-021-04332-2

[B22] Edwards, R.A., and Rohwer, F. (2005) Viral metagenomics. Nat Rev Microbiol 3: 504–510. 15886693 10.1038/nrmicro1163

[B23] Fukasawa, F., Hirai, M., Takaki, Y., Shimane, Y., Thomas, C.E., Urayama, S., et al. (2020) A new polycipivirus identified in Colobopsis shohki. Arch Virol 165: 761–763. 31865471 10.1007/s00705-019-04510-8

[B24] Ghabrial, S.A., Castón, J.R., Jiang, D., Nibert, M.L., and Suzuki, N. (2015) 50-plus years of fungal viruses. Virology 479: 356–368. 25771805 10.1016/j.virol.2015.02.034

[B25] Ghosh, S., and Malik, Y.S. (2021) The true host/s of picobirnaviruses. Front Vet Sci 7: 615293. 33553283 10.3389/fvets.2020.615293 PMC7855169

[B26] Goodman, R.P., Ghabrial, S.A., Fichorova, R.N., and Nibert, M.L. (2011) Trichomonasvirus: a new genus of protozoan viruses in the family Totiviridae. Arch Virol 156: 171–179. 20976609 10.1007/s00705-010-0832-8 PMC3659425

[B27] Hillman, B.I., and Cai, G. (2013) The family Narnaviridae: simplest of RNA viruses. Adv Virus Res 86: 149–176. 23498906 10.1016/B978-0-12-394315-6.00006-4

[B28] Hirai, M., Takaki, Y., Kondo, F., Horie, M., Urayama, S., and Nunoura, T. (2021) RNA viral metagenome ana­lysis of subnanogram dsRNA using fragmented and primer ligated dsRNA sequencing (FLDS). Microbes Environ 36: ME20152. 33952860 10.1264/jsme2.ME20152 PMC8209451

[B29] Huang, Y., Niu, B., Gao, Y., Fu, L., and Li, W. (2010) CD-HIT Suite: a web server for clustering and comparing biological sequences. Bioinformatics 26: 680–682. 20053844 10.1093/bioinformatics/btq003 PMC2828112

[B30] Izumi, T., Morioka, Y., Urayama, S., Motooka, D., Tamura, T., Kawagishi, T., et al. (2021) DsRNA sequencing for RNA virus surveillance using human clinical samples. Viruses 13: 1310. 34372516 10.3390/v13071310 PMC8309968

[B31] Kaneko, H., Blanc-Mathieu, R., Endo, H., Chaffron, S., Delmont, T.O., Gaia, M., et al. (2021) Eukaryotic virus composition can predict the efficiency of carbon export in the global ocean. iScience 24: 102002. 33490910 10.1016/j.isci.2020.102002 PMC7811142

[B32] Keel, B.N., Deng, B., and Moriyama, E.N. (2018) MOCASSIN-prot: a multi-objective clustering approach for protein similarity networks. Bioinformatics 34: 1270–1277. 29186344 10.1093/bioinformatics/btx755

[B33] Khoshnan, A., and Alderete, J. (1994) Trichomonas vaginalis with a double-stranded RNA virus has upregulated levels of phenotypically variable immunogen mRNA. J Virol 68: 4035–4038. 8189538 10.1128/jvi.68.6.4035-4038.1994 PMC236912

[B34] King, A.M.Q., Adams, M.J., Carstens, E.B., and Lefkowitz, E.J. (2012) *Virus Taxonomy: Classification and Nomenclature of Viruses: Ninth Report of the International Committee on Taxonomy of Viruses*. Amsterdam: Elsevier.

[B35] Koonin, E.V., Dolja, V.V., and Krupovic, M. (2015) Origins and evolution of viruses of eukaryotes: The ultimate modularity. Virology 479–480: 2–25. 10.1016/j.virol.2015.02.039 PMC5898234 25771806

[B36] Koonin, E.V., Dolja, V.V., Krupovic, M., Varsani, A., Wolf, Y.I., Yutin, N., et al. (2020) Global organization and proposed megataxonomy of the virus world. Microbiol Mol Biol Rev 84: e00061-00019. 32132243 10.1128/MMBR.00061-19 PMC7062200

[B37] Kotta-Loizou, I., and Coutts, R.H. (2017) Mycoviruses in Aspergilli: a comprehensive review. Front Microbiol 8: 1699. 28932216 10.3389/fmicb.2017.01699 PMC5592211

[B38] Lachnit, T., Thomas, T., and Steinberg, P. (2016) Expanding our understanding of the seaweed holobiont: RNA viruses of the red alga Delisea pulchra. Front Microbiol 6: 1489. 26779145 10.3389/fmicb.2015.01489 PMC4705237

[B39] Lang, A.S., Culley, A.I., and Suttle, C.A. (2004) Genome sequence and characterization of a virus (HaRNAV) related to picorna-like viruses that infects the marine toxic bloom-forming alga Heterosigma akashiwo. Virology 320: 206–217. 15016544 10.1016/j.virol.2003.10.015

[B41] Lang, A.S., Vlok, M., Culley, A.I., Suttle, C.A., Takao, Y., Tomaru, Y., and ICTV Report Consortium (2021) ICTV virus taxonomy profile: Marnaviridae 2021. J Gen Virol 102: 001633. 34356002 10.1099/jgv.0.001633 PMC8513639

[B42] Liu, Y.C., Linder-Basso, D., Hillman, B.I., Kaneko, S., and Milgroom, M.G. (2003) Evidence for interspecies transmission of viruses in natural populations of filamentous fungi in the genus Cryphonectria. Mol Ecol 12: 1619–1628. 12755889 10.1046/j.1365-294x.2003.01847.x

[B43] Loeffler, F., and Frosch, P. (1898) Report of the commission for research on foot-and-mouth disease. Zent Bakt Parasitkde Abt I 23: 371–391.

[B44] Marquez, L.M., Redman, R.S., Rodriguez, R.J., and Roossinck, M.J. (2007) A virus in a fungus in a plant: three-way symbiosis required for thermal tolerance. Science 315: 513–515. 17255511 10.1126/science.1136237

[B45] Márquez, L.M., and Roossinck, M.J. (2012) Do persistent RNA viruses fit the trade-off hypothesis of virulence evolution? Curr Opin Virol 2: 556–560. 22819020 10.1016/j.coviro.2012.06.010

[B46] Mihara, T., Nishimura, Y., Shimizu, Y., Nishiyama, H., Yoshikawa, G., Uehara, H., et al. (2016) Linking virus genomes with host taxonomy. Viruses 8: 66. 26938550 10.3390/v8030066 PMC4810256

[B47] Morris, T., and Dodds, J. (1979) Isolation and ana­lysis of double-stranded RNA from virus-infected plant and fungal tissue. Phytopathology 69: 854–858. 10.1094/Phyto-69-854 40934407

[B48] Nasir, A., Forterre, P., Kim, K.M., and Caetano-Anollés, G. (2014) The distribution and impact of viral lineages in domains of life. Front Microbiol 5: 194. 24817866 10.3389/fmicb.2014.00194 PMC4012193

[B49] Nerva, L., Ciuffo, M., Vallino, M., Margaria, P., Varese, G.C., Gnavi, G., and Turina, M. (2016) Multiple approaches for the detection and characterization of viral and plasmid symbionts from a collection of marine fungi. Virus Res 219: 22–38. 26546154 10.1016/j.virusres.2015.10.028

[B50] Nibert, M.L., Ghabrial, S.A., Maiss, E., Lesker, T., Vainio, E.J., Jiang, D., and Suzuki, N. (2014) Taxonomic reorganization of family Partitiviridae and other recent progress in partitivirus research. Virus Res 188: 128–141. 24768846 10.1016/j.virusres.2014.04.007

[B51] Ninomiya, A., Urayama, S., Suo, R., Itoi, S., Fuji, S.I., Moriyama, H., and Hagiwara, D. (2020) Mycovirus-induced tenuazonic acid production in a rice blast fungus Magnaporthe oryzae. Front Microbiol 11: 1641. 32765467 10.3389/fmicb.2020.01641 PMC7379127

[B52] Nuss, D.L. (2005) Hypovirulence: mycoviruses at the fungal-plant interface. Nat Rev Microbiol 3: 632–642. 16064055 10.1038/nrmicro1206

[B53] Obeng, N., Pratama, A.A., and Elsas, J.D.V. (2016) The significance of mutualistic phages for bacterial ecology and evolution. Trends Microbiol 24: 440–449. 26826796 10.1016/j.tim.2015.12.009

[B54] Okada, R., Ichinose, S., Takeshita, K., Urayama, S., Fukuhara, T., Komatsu, K., et al. (2018) Molecular characterization of a novel mycovirus in Alternaria alternata manifesting two-sided effects: Down-regulation of host growth and up-regulation of host plant pathogenicity. Virology 519: 23–32. 29631173 10.1016/j.virol.2018.03.027

[B55] Pratama, A.A., and van Elsas, J.D. (2018) The ‘neglected’ soil virome—Potential role and impact. Trends Microbiol 26: 649–662. 29306554 10.1016/j.tim.2017.12.004

[B56] Reed, W., and Carroll, J. (1901) The prevention of yellow fever. Public Health Pap Rep 27: 113–129. 19600973 PMC2329408

[B57] Roossinck, M.J. (2010) Lifestyles of plant viruses. Philos Trans R Soc Lond B Biol Sci 365: 1899–1905. 20478885 10.1098/rstb.2010.0057 PMC2880111

[B58] Roossinck, M.J., Saha, P., Wiley, G.B., Quan, J., White, J.D., Lai, H., et al. (2010) Ecogenomics: using massively parallel pyrosequencing to understand virus ecology. Mol Ecol 19 Suppl 1: 81–88. 20331772 10.1111/j.1365-294X.2009.04470.x

[B59] Roossinck, M.J. (2011) The good viruses: viral mutualistic symbioses. Nat Rev Microbiol 9: 99–108. 21200397 10.1038/nrmicro2491

[B60] Roossinck, M.J. (2012) Persistent plant viruses: Molecular hitchhikers or epigenetic elements? In *Viruses: Essential Agents of Life*. Witzany, G. (ed.) Dordrecht: Springer Netherlands, pp. 177–186.

[B61] Roossinck, M.J. (2015) Metagenomics of plant and fungal viruses reveals an abundance of persistent lifestyles. Front Microbiol 5: 767. 25628611 10.3389/fmicb.2014.00767 PMC4290624

[B62] Roossinck, M.J. (2019) Evolutionary and ecological links between plant and fungal viruses. New Phytol 221: 86–92. 30084143 10.1111/nph.15364

[B63] Sadeghi, M., Tomaru, Y., and Ahola, T. (2021) RNA viruses in aquatic unicellular eukaryotes. Viruses 13: 362. 33668994 10.3390/v13030362 PMC7996518

[B64] Schmitt, M.J., and Breinig, F. (2002) The viral killer system in yeast: from mole­cular biology to application. FEMS Microbiol Rev 26: 257–276. 12165427 10.1111/j.1574-6976.2002.tb00614.x

[B65] Shi, M., Lin, X.D., Tian, J.H., Chen, L.J., Chen, X., Li, C.X., et al. (2016) Redefining the invertebrate RNA virosphere. Nature 540: 539–543. 27880757 10.1038/nature20167

[B66] Starr, E.P., Nuccio, E.E., Pett-Ridge, J., Banfield, J.F., and Firestone, M.K. (2019) Metatranscriptomic reconstruction reveals RNA viruses with the potential to shape carbon cycling in soil. Proc Natl Acad Sci U S A 116: 25900–25908. 31772013 10.1073/pnas.1908291116 PMC6926006

[B67] Steward, G.F., Culley, A.I., Mueller, J.A., Wood-Charlson, E.M., Belcaid, M., and Poisson, G. (2013) Are we missing half of the viruses in the ocean? ISME J 7: 672–679. 23151645 10.1038/ismej.2012.121 PMC3578568

[B68] Stuart, K.D., Weeks, R., Guilbride, L., and Myler, P.J. (1992) Molecular organization of Leishmania RNA virus 1. Proc Natl Acad Sci U S A 89: 8596–8600. 1382295 10.1073/pnas.89.18.8596 PMC49967

[B69] Suttle, C.A. (2016) Environmental microbiology: Viral diversity on the global stage. Nat Microbiol 1: 16205. 27782140 10.1038/nmicrobiol.2016.205

[B70] Suzuki, Y., Baidaliuk, A., Miesen, P., Frangeul, L., Crist, A.B., Merkling, S.H., et al. (2020) Non-retroviral endogenous viral element limits cognate virus replication in Aedes aegypti ovaries. Curr Biol 30: 3495–3506.e6. 32679098 10.1016/j.cub.2020.06.057 PMC7522710

[B71] Tai, V., Lawrence, J.E., Lang, A.S., Chan, A.M., Culley, A.I., and Suttle, C.A. (2003) Characterization of HaRNAV, a single‐stranded RNA virus causing lysis of Heterosigma akashiwo (raphidophyceae) 1. J Phycol 39: 343–352.

[B72] Takahashi-Nakaguchi, A., Shishido, E., Yahara, M., Urayama, S., Sakai, K., Chibana, H., et al. (2019) Analysis of an intrinsic mycovirus associated with reduced virulence of the human pathogenic fungus Aspergillus fumigatus. Front Microbiol 10: 3045. 32010101 10.3389/fmicb.2019.03045 PMC6978690

[B73] Takao, Y., Nagasaki, K., Mise, K., Okuno, T., and Honda, D. (2005) Isolation and characterization of a novel single-stranded RNA virus infectious to a marine fungoid protist, Schizochytrium sp. (Thraustochytriaceae, Labyrinthulea). Appl Environ Microbiol 71: 4516–4522. 16085844 10.1128/AEM.71.8.4516-4522.2005 PMC1183295

[B74] Takao, Y., Mise, K., Nagasaki, K., Okuno, T., and Honda, D. (2006) Complete nucleotide sequence and genome organization of a single-stranded RNA virus infecting the marine fungoid protist Schizochytrium sp. J Gen Virol 87: 723–733. 16476996 10.1099/vir.0.81204-0

[B75] Tomaru, Y., Katanozaka, N., Nishida, K., Shirai, Y., Tarutani, K., Yamaguchi, M., and Nagasaki, K. (2004) Isolation and characterization of two distinct types of HcRNAV, a single-stranded RNA virus infecting the bivalve-killing microalga Heterocapsa circularisquama. Aquat Microb Ecol 34: 207–218.

[B76] Torrecilhas, A.C., Soares, R.P., Schenkman, S., Fernandez-Prada, C., and Olivier, M. (2020) Extracellular vesicles in trypanosomatids: host cell communication. Front Cell Infect Microbiol 10: 602502. 33381465 10.3389/fcimb.2020.602502 PMC7767885

[B77] Uchida, K., Sakuta, K., Ito, A., Takahashi, Y., Katayama, Y., Omatsu, T., et al. (2021) Two novel endornaviruses co-infecting a phytophthora pathogen of Asparagus officinalis modulate the developmental stages and fungicide sensitivities of the host oomycete. Front Microbiol 12: 122. 10.3389/fmicb.2021.633502 PMC7902037 33633714

[B78] Uehara-Ichiki, T., Urayama, S., Hirai, M., Takaki, Y., Nunoura, T., Fujinaga, M., and Hanada, K. (2021) Complete genome sequence of Sikte (Sitke) waterborne virus, a member of the genus Tombusvirus. Arch Virol 166: 991–994. 33492526 10.1007/s00705-020-04949-0

[B79] Urayama, S., Takaki, Y., and Nunoura, T. (2016) FLDS: A comprehensive dsRNA sequencing method for intracellular RNA virus surveillance. Microbes Environ 31: 33–40. 26877136 10.1264/jsme2.ME15171 PMC4791113

[B80] Urayama, S., Takaki, Y., Nishi, S., Yoshida-Takashima, Y., Deguchi, S., Takai, K., and Nunoura, T. (2018a) Unveiling the RNA virosphere associated with marine microorganisms. Mol Ecol Resour 18: 1444–1455. 30256532 10.1111/1755-0998.12936

[B81] Urayama, S., Takaki, Y., Nunoura, T., and Miyamoto, N. (2018b) Complete genome sequence of a novel RNA virus identified from a deep-sea animal, Osedax japonicus. Microbes Environ 33: 446–449. 30318497 10.1264/jsme2.ME18089 PMC6308001

[B82] Urayama, S., Doi, N., Kondo, F., Chiba, Y., Takaki, Y., Hirai, M., et al. (2020a) Diverged and active partitiviruses in lichen. Front Microbiol 11: 561344. 33193146 10.3389/fmicb.2020.561344 PMC7609399

[B83] Urayama, S., Takaki, Y., Hagiwara, D., and Nunoura, T. (2020b) dsRNA-seq reveals novel RNA virus and virus-like putative complete genome sequences from Hymeniacidon sp. sponge. Microbes Environ 35: ME19132. 32115438 10.1264/jsme2.ME19132 PMC7308569

[B84] Vainio, E.J., Hakanpää, J., Dai, Y.-C., Hansen, E., Korhonen, K., and Hantula, J. (2011) Species of Heterobasidion host a diverse pool of partitiviruses with global distribution and interspecies transmission. Fungal Biol 115: 1234–1243. 22115442 10.1016/j.funbio.2011.08.008

[B85] van der Lende, T.R., Harmsen, M.C., and Wessels, J.G. (1994) Double-stranded RNAs and proteins associated with the 34‍ ‍nm virus particles of the cultivated mushroom Agaricus bisporus. J Gen Virol 75: 2533–2536. 8077959 10.1099/0022-1317-75-9-2533

[B86] Wang, A.L., and Wang, C.C. (1986) Discovery of a specific double-stranded RNA virus in Giardia lamblia. Mol Biochem Parasitol 21: 269–276. 3807947 10.1016/0166-6851(86)90132-5

[B87] Wickner, R.B. (1996) Double-stranded RNA viruses of Saccharomyces cerevisiae. Microbiol Rev 60: 250–265. 8852903 10.1128/mr.60.1.250-265.1996 PMC239427

[B88] Wilhelm, S.W., and Suttle, C.A. (1999) Viruses and nutrient cycles in the sea: viruses play critical roles in the structure and function of aquatic food webs. Bioscience 49: 781–788.

[B89] Wolf, Y.I., Silas, S., Wang, Y., Wu, S., Bocek, M., Kazlauskas, D., et al. (2020) Doubling of the known set of RNA viruses by metagenomic ana­lysis of an aquatic virome. Nat Microbiol 5: 1262–1270. 32690954 10.1038/s41564-020-0755-4 PMC7508674

[B90] Xu, P., Chen, F., Mannas, J.P., Feldman, T., Sumner, L.W., and Roossinck, M.J. (2008) Virus infection improves drought tolerance. New Phytol 180: 911–921. 18823313 10.1111/j.1469-8137.2008.02627.x

[B91] Zayed, A.A., Wainaina, J.M., Dominguez-Huerta, G., Pelletier, E., Guo, J., Mohssen, M., et al. (2022) Cryptic and abundant marine viruses at the evolutionary origins of Earth’s RNA virome. Science 376: 156–162. 35389782 10.1126/science.abm5847 PMC10990476

[B92] Zhang, R., Liu, S., Chiba, S., Kondo, H., Kanematsu, S., and Suzuki, N. (2014) A novel single-stranded RNA virus isolated from a phytopathogenic filamentous fungus, Rosellinia necatrix, with similarity to hypo-like viruses. Front Microbiol 5: 360. 25101066 10.3389/fmicb.2014.00360 PMC4103508

[B93] Zimmerman, A.E., Howard-Varona, C., Needham, D.M., John, S.G., Worden, A.Z., Sullivan, M.B., et al. (2020) Metabolic and biogeochemical consequences of viral infection in aquatic ecosystems. Nat Rev Microbiol 18: 21–34. 31690825 10.1038/s41579-019-0270-x

