# Supplementary material for: Eukaryotic Microbial RNA Viruses—Acute or Persistent? Insights into Their Function in the Aquatic Ecosystem
Source: Microbes Environ. 2022 Aug 3;37(5):ME22034. doi: 10.1264/jsme2.ME22034 (PMC9763035; doi:10.1264/jsme2.ME22034)
Supplement: Supplementary file 1 — Supplementary Material [file 37_22034_s1.pdf]

acute/persistent	reference	virus tax id	virus name	refseq id	host tax id	host name	pmid	taxonomic group
acute	Comparison of genome sequences of single-stranded RNA viruses infecting the bivalve-killing dinoflagellate Heterocapsa	2030964	Heterocapsa circularisquama RNA virus 01	NC_007518	139025	Heterocapsa circularisquama		Alveolata
acute	Complete nucleotide sequence and genome organization of a single-stranded RNA virus infecting the marine fungoid protist Schizochytrium sp.	674971	Aurantiochytrium single-stranded RNA virus 01	NC_007522	1907177	Schizochytrium sp.	16476996	Stramenopiles
acute	Isolation and characterization of a single-stranded RNA virus infecting the bloom-forming diatom Chaetoceros socialis	2169725	Chaetoceros socialis forma radians RNA virus 1	NC_012212	163503	Chaetoceros socialis	19233955	Stramenopiles
acute	Isolation and Characterization of a Single-Stranded RNA Virus Infecting the Marine Planktonic Diatom Chaetoceros tenuissimus Meunier	497136	Chaetoceros tenuissimus RNA virus 01	NC_038321	426638	Chaetoceros tenuissimus		Stramenopiles
acute	Isolation and characterization of a single-stranded RNA virus that infects the marine planktonic diatom Chaetoceros sp. (SS08-C03)	1045800	Chaetoceros species RNA virus 02	NC_055125	49240	Chaetoceros sp.		Stramenopiles
acute	Discovery of Two Novel Viruses Expands the Diversity of Single-Stranded DNA and Single-Stranded RNA Viruses Infecting a Cosmopolitan Marine Diatom	1516128	Chaetoceros tenuissimus RNA virus type-II	NC_025889	49240	Chaetoceros sp.	25452289	Stramenopiles
acute	Isolation and Characterization of a Novel Single-Stranded RNA Virus Infecting the Bloom-Forming Diatom Rhizosolenia setigera	359987	Rhizosolenia setigera RNA virus 01	NC_018613	3005	Rhizosolenia setigera		Stramenopiles
acute	First evidence for the existence of pennate diatom viruses	1522179	Asterionellopsis glacialis RNA virus	NC_024489	33640	Asterionellopsis glacialis	22237541	Stramenopiles
acute	CHARACTERIZATION OF HaRNAV, A SINGLE-STRANDED RNA VIRUS CAUSING LYSIS OF HETEROSIGMA AKASHIWO (RAPHIDOPHYCEAE)	324900	Heterosigma akashiwo RNA virus	NC_005281	2829	Heterosigma akashiwo		Stramenopiles
acute	Micromonas pusilla reovirus: a new member of the family Reoviridae assigned to a novel proposed genus (Mimoreovirus)	338781	Micromonas pusilla reovirus	NC_008171, NC_008172, NC_008173, NC_008174, NC_008175, NC_008176, NC_008177, NC_008178, NC_008179, NC_008180, NC_008181	38833	Micromonas pusilla		Green Algae
persistent		2364200	Blechmonas luni narnavirus 1	NC_040829	1463349	Blechomonas luni	30327446	Trypanosomatidae
persistent		2364202	Blechomonas maslovi narnavirus 1	NC_040571	1463231	Blechomonas maslovi	30327446	Trypanosomatidae
persistent		2364201	Blechomonas wendygibsoni narnavirus 1	NC_040641	1463346	Blechomonas wendygibsoni		Trypanosomatidae
persistent		1497019	Leishmania aethiopica RNA virus	NC_024115	5667	Leishmania aethiopica		Trypanosomatidae
persistent		58103	Leishmania RNA virus 1 - 1	NC_002063	5664	Leishmania major		Trypanosomatidae
persistent		39116	Leishmania RNA virus 2 - 1	NC_002064	5664	Leishmania major		Trypanosomatidae
persistent		12530	Leishmania RNA virus 1 - 4	NC_003601	87235	Leishmania braziliensis guyanensis		Trypanosomatidae
persistent		1856767	Leptomonas seymouri Narna-like virus 1	NC_040764, NC_040765	5684	Leptomonas seymouri		Trypanosomatidae
persistent		1859148	Leptomonas moramango leishbunyavirus	NC_055202, NC_055203, NC_055204	1503871	Leptomonas moramango		Trypanosomatidae
persistent	A Narnavirus in the Trypanosomatid Protist Plant Pathogen Phytomonas serpens	1856627	Phytomonas serpens narnavirus 1	NC_030308	1276585	Phytomonas serpens 9T		Trypanosomatidae
persistent	Giardia canis: ultrastructural analysis of G. canis trophozoites transfected with full length G. canis virus cDNA transcripts	353633	Giardia canis virus	DQ238861	5740	Giardia		Flagellate
persistent		29255	Giardia lamblia virus	NC_003555	5741	Giardia intestinalis		Flagellate
persistent		674953	Trichomonas vaginalis virus 1	NC_027701	5722	Trichomonas vaginalis		Flagellate
persistent		674954	Trichomonas vaginalis virus 2	NC_003873	5722	Trichomonas vaginalis		Flagellate
persistent		170965	Trichomonas vaginalis virus 3	NC_004034	5722	Trichomonas vaginalis	21110050	Flagellate
persistent		1008292	Trichomonas vaginalis virus 4	NC_038700	5722	Trichomonas vaginalis		Flagellate
persistent		29256	Trichomonas vaginalis virus	NC_003824	5722	Trichomonas vaginalis	7831841	Flagellate
persistent	Complete Genome of a Novel Endornavirus Assembled from Next-Generation Sequence Data	1249676	Grapevine endophyte alphaendornavirus	NC_019493	4751	Fungi	23118465	
persistent		1405299	Botryosphaeria dothidea chrysovirus 1	NC_034268, NC_034269, NC_034270, NC_034271	55169	Botryosphaeria dothidea		Fungi
persistent		1547580	Botryosphaeria dothidea victorivirus 1	NC_025214	55169	Botryosphaeria dothidea		Fungi
persistent		1516075	Botryosphaeria dothidea virus 1	NC_033476, NC_033477, NC_033494, NC_033495,	55169	Botryosphaeria dothidea		Fungi
persistent		1740646	Beauveria bassiana polymycovirus 1	NC_034257, NC_034258, NC_034259, NC_034260	55169	Botryosphaeria dothidea	28114361	Fungi
persistent		459770	Diplodia scrobiculata RNA virus 1	NC_013699	280322	Diplodia scrobiculata		Fungi
persistent		73497	Sphaeropsis sapinea RNA virus 1	NC_001963	66738	Diplodia sapinea	9878619	Fungi
persistent		73498	Sphaeropsis sapinea RNA virus 2	NC_001964	66738	Diplodia sapinea	9878619	Fungi
persistent		1708483	Macrophomina phaseolina chrysovirus 1	NC_043662, NC_043663, NC_043664, NC_043665	35725	Macrophomina phaseolina		Fungi
persistent		1527523	Macrophomina phaseolina tobamo-like virus	NC_025674	35725	Macrophomina phaseolina		Fungi
persistent		2587541	Neofusicoccum parvum chrysovirus 1	NC_055628, NC_055629, NC_055630, NC_055631	310453	Neofusicoccum parvum		Fungi
persistent		1985159	Neofusicoccum luteum mitovirus 1	NC_035114	120395	Neofusicoccum luteum		Fungi
persistent		1985188	Neofusicoccum luteum fusarivirus 1	NC_040791	120395	Neofusicoccum luteum		Fungi
persistent		210827	Coniothyrium minitans RNA virus	NC_007523	565426	Paraphaeosphaeria minitans	12727341	Fungi
persistent		2163916	Leptosphaeria biglobosa mitovirus 1	NC_040819	220672	Leptosphaeria biglobosa		Fungi
persistent		1532031	Alternaria longipes dsRNA virus 1	NC_024703	160389	Alternaria longipes		Fungi
persistent		483537	Alternaria alternata virus 1	NC_010984, NC_010989, NC_010990, NC_010991	5599	Alternaria alternata	19118588	Fungi
persistent		2066695	Alternaria alternata chrysovirus 1	NC_040736, NC_040737, NC_040738, NC_040739,	5599	Alternaria alternata	29631173	Fungi
persistent		1728964	Alternaria arborescens victorivirus 1	NC_040793	1122867	Alternaria arborescens EGS 39-128	26923927	Fungi
persistent		1826822	Alternaria arborescens mitovirus 1	NC_030747	1122867	Alternaria arborescens EGS 39-128	27368994	Fungi
persistent		1580606	Alternaria brassicicola betaendornavirus 1	NC_026136	29001	Alternaria brassicicola		Fungi
persistent		2138326	Bipolaris maydis botybirnavirus 1	NC_040395, NC_040396	5016	Bipolaris maydis	29967958	Fungi
persistent		164750	Helminthosporium victoriae 145S virus	NC_005978, NC_005979, NC_005980, NC_005981	40125	Bipolaris victoriae	664249	Fungi
persistent		45237	Helminthosporium victoriae virus 190S	NC_003607	40125	Bipolaris victoriae	664249	Fungi
persistent		1980631	Bipolaris maydis partitivirus 1	NC_034514, NC_034524	5016	Bipolaris maydis		Fungi
persistent		2483307	Bipolaris maydis partitivirus 2	NC_040495, NC_040496, NC_040497	5016	Bipolaris maydis		Fungi

persistent		421976	Curvularia thermal tolerance virus	NC_010985, NC_010986	671092	Curvularia protuberata	17255511	Fungi
persistent		1755785	Pleospora typhicola fusarivirus 1	NC_028470	1075110	Pleospora typhicola		Fungi
persistent		1087068	Aspergillus foetidus dsRNA mycovirus	NC_020100, NC_020101, NC_020102, NC_020103	63131	Aspergillus foetidus		Fungi
persistent		2164061	Aspergillus thermomutatus chrysovirus 1	NC_055605, NC_055606, NC_055607, NC_055608	41047	Aspergillus thermomutatus	30571748	Fungi
persistent		1087070	Aspergillus foetidus slow virus 1	NC_038928	63131	Aspergillus foetidus	22729614	Fungi
persistent		216371	Penicillium stoloniferum virus S	NC_005976, NC_005977	40380	Aspergillus ochraceus	14618085	Fungi
persistent		607716	Aspergillus fumigatus chrysovirus	NC_038871, NC_038872, NC_038873, NC_038874	746128	Aspergillus fumigatus	20621139	Fungi
persistent		2250452	Aspergillus fumigatus partitivirus 2	NC_040756, NC_040757	746128	Aspergillus fumigatus	30044844	Fungi
persistent		2250469	Aspergillus fumigatus polymycovirus 1	NC_040418, NC_040419, NC_040420, NC_040421	746128	Aspergillus fumigatus	30044844	Fungi
persistent		158372	Penicillium chrysogenum virus	NC_007539, NC_007540, NC_007541, NC_007542	5076	Penicillium chrysogenum		Fungi
persistent		2682569	Chrysothrix chrysovirus 1	NC_055654, NC_055655, NC_055656, NC_055657	1651864	Penicillium citreosulfuratum		Fungi
persistent		1755792	Penicillium janczewskii chrysovirus 1	NC_028495, NC_028496, NC_028497, NC_028500	121612	Penicillium janczewskii		Fungi
persistent		1755793	Penicillium janczewskii chrysovirus 2	NC_043669, NC_043670, NC_043671, NC_043672	121612	Penicillium janczewskii		Fungi
persistent		1755467	Penicillium aurantiogriseum totivirus 1	NC_028948	36655	Penicillium aurantiogriseum		Fungi
persistent		1833938	Penicillium digitatum virus 1	NC_029989	36651	Penicillium digitatum		Fungi
persistent		2164100	Penicillium digitatum narna-like virus 1	NC_040683	36651	Penicillium digitatum		Fungi
persistent		296210	Penicillium stoloniferum virus F	NC_007221, NC_007222	1343417	Penicillium stoloniferum	16025243	Fungi
persistent		1756157	Penicillium aurantiogriseum partitivirus 1	NC_028494, NC_028499	36655	Penicillium aurantiogriseum		Fungi
persistent		2164101	Penicillium digitatum polymycoviruses 1	NC_040422, NC_040423, NC_040424, NC_040425	36651	Penicillium digitatum	29615698	Fungi
persistent		2485923	Penicillium brevicompactum tetramycovirus 1	NC_055286, NC_055287, NC_055288, NC_055289	5074	Penicillium brevicompactum		Fungi
persistent		1755752	Penicillium aurantiogriseum fusarivirus 1	NC_028467	36655	Penicillium aurantiogriseum		Fungi
persistent		1532180	Penicillium roqueforti ssRNA mycovirus 1	NC_024699	5082	Penicillium roqueforti		Fungi
persistent		2052561	Erysiphe necator mitovirus 1	NC_037054	52586	Erysiphe necator		Fungi
persistent		2052562	Erysiphe necator mitovirus 2	NC_037055	52586	Erysiphe necator		Fungi
persistent		2052563	Erysiphe necator mitovirus 3	NC_037056	52586	Erysiphe necator		Fungi
persistent	Complete sequence of a double-stranded RNA from the phytopathogenic fungus Erysiphe cichoracearum that might represent a novel endornavirus	1777015	Erysiphe cichoracearum alphaendornavirus	NC_029095	62708	Golovinomyces cichoracearum		Fungi
persistent		279539	Gremmeniella abietina RNA virus L2	NC_005965	127520	Gremmeniella abietina		Fungi
persistent		152217	Gremmeniella abietina RNA virus L1	NC_003876	127520	Gremmeniella abietina		Fungi
persistent		374005	Gremmeniella abietina type B RNA virus XL1	NC_007920	127520	Gremmeniella abietina		Fungi
persistent		374006	Gremmeniella abietina type B RNA virus XL2	DQ399290	127520	Gremmeniella abietina		Fungi
persistent		279538	Gremmeniella abietina mitochondrial RNA virus S2	NC_006264	127520	Gremmeniella abietina		Fungi
persistent		191436	Gremmeniella abietina RNA virus MS1	NC_004018, NC_004019, NC_004020	127520	Gremmeniella abietina		Fungi
persistent		1872719	Botrytis ourmia-like virus	NC_028476	33196	Botrytis		Fungi
persistent		1568973	Botrytis cinerea RNA virus 1	NC_026139	40559	Botrytis cinerea	25595766	Fungi
persistent		1918014	Botrytis porri botybirnavirus 1	NC_017990, NC_017991	87229	Botrytis porri		Fungi
persistent		425009	Botryotinia fuckeliana totivirus 1	NC_009224	40559	Botrytis cinerea		Fungi
persistent		2169747	Botrytis cinerea betaendornavirus 1	NC_031752	40559	Botrytis cinerea		Fungi
persistent		174142	Botrytis virus X	NC_005132	40559	Botrytis cinerea		Fungi
persistent		129395	Botrytis virus F	NC_002604	40559	Botrytis cinerea		Fungi
persistent		444193	Botrytis cinerea mitovirus 1	NC_011372	40559	Botrytis cinerea		Fungi
persistent		1629665	Botrytis cinerea mitovirus 2	NC_028471	40559	Botrytis cinerea		Fungi
persistent		1629666	Botrytis cinerea mitovirus 3	NC_028472	40559	Botrytis cinerea		Fungi
persistent		2219105	Botrytis cinerea hypovirus 1	NC_037659	40559	Botrytis cinerea	29757259	Fungi
persistent		425010	Botryotinia fuckeliana partitivirus 1	NC_010349, NC_010350, NC_010351	40559	Botrytis cinerea		Fungi
persistent		2219106	Botrytis cinerea fusarivirus 1	NC_037660	40559	Botrytis cinerea	29757259	Fungi
persistent		2219107	Botrytis cinerea fusarivirus 1-S1	NC_037661	40559	Botrytis cinerea	29757259	Fungi
persistent		2219108	Botrytis cinerea fusarivirus 1-S2	NC_037662	40559	Botrytis cinerea	29757259	Fungi
persistent		1629671	Botrytis cinerea negative-stranded RNA virus 1	NC_028466	40559	Botrytis cinerea	27685856	Fungi
persistent		1181310	Sclerotinia sclerotiorum dsRNA mycovirus-L	NC_017915	5180	Sclerotinia sclerotiorum		Fungi
persistent		1654339	Sclerotinia sclerotiorum botybirnavirus 1	NC_027138, NC_027139	5180	Sclerotinia sclerotiorum		Fungi
persistent		1661257	Sclerotinia sclerotiorum megabirnavirus 1	NC_027221, NC_027222	5180	Sclerotinia sclerotiorum		Fungi
persistent		1859161	Sclerotinia nivalis victorivirus 1	NC_030392	352851	Sclerotinia nivalis		Fungi
persistent		1840528	Sclerotinia sclerotiorum mycoreovirus 4	NC_030152, NC_030153, NC_030154, NC_030155, NC_030156, NC_030157, NC_030158, NC_030159, NC_030160, NC_030161, NC_030162, NC_030163	5180	Sclerotinia sclerotiorum		Fungi
persistent		2003398	Sclerotinia sclerotiorum betaendornavirus 1	NC_023893	5180	Sclerotinia sclerotiorum		Fungi
persistent		2316144	Sclerotinia minor endornavirus 1	NC_040631	38451	Sclerotinia minor		Fungi
persistent		1353016	Sclerotinia sclerotiorum endornavirus 1	NC_021706	5180	Sclerotinia sclerotiorum		Fungi
persistent		1788309	Sclerotinia sclerotiorum deltaflexivirus 1	NC_038977	5180	Sclerotinia sclerotiorum		Fungi
persistent		2219092	Sclerotinia sclerotiorum deltaflexivirus 2	NC_040649	5180	Sclerotinia sclerotiorum		Fungi
persistent		1435450	Sclerotinia sclerotiorum umbra-like virus 1	NC_030203	5180	Sclerotinia sclerotiorum		Fungi
persistent		1133727	Sclerotinia sclerotiorum mitovirus 1	JQ013377	5180	Sclerotinia sclerotiorum		Fungi
persistent		1133728	Sclerotinia sclerotiorum mitovirus 2	NC_040434	5180	Sclerotinia sclerotiorum		Fungi
persistent		1279099	Sclerotinia sclerotiorum mitovirus 3	NC_028475	5180	Sclerotinia sclerotiorum		Fungi
persistent		1435446	Sclerotinia sclerotiorum mitovirus 6	NC_023598	5180	Sclerotinia sclerotiorum		Fungi
persistent		1494074	Sclerotinia sclerotiorum mitovirus 1 HC025	NC_026510	5180	Sclerotinia sclerotiorum		Fungi
persistent		1708390	Sclerotinia sclerotiorum ourmia-like virus 2	NC_055145	5180	Sclerotinia sclerotiorum		Fungi
persistent		1708389	Sclerotinia sclerotiorum ourmia-like virus 1	NC_055144	5180	Sclerotinia sclerotiorum		Fungi
persistent		1708391	Sclerotinia sclerotiorum negative-stranded RNA virus 4	NC_043483	5180	Sclerotinia sclerotiorum		Fungi

persistent	1483724	Sclerotinia sclerotiorum negative-stranded RNA virus 1	NC_025383	5180	Sclerotinia sclerotiorum		Fungi
persistent	1435457	Sclerotinia sclerotiorum negative-stranded RNA virus 3	NC_026732	5180	Sclerotinia sclerotiorum		Fungi
persistent	1074325	Sclerotinia sclerotiorum hypovirus 1	NC_015939	5180	Sclerotinia sclerotiorum		Fungi
persistent	1423347	Sclerotinia sclerotiorum hypovirus 2	NC_022896	5180	Sclerotinia sclerotiorum		Fungi
persistent	659497	Sclerotinia sclerotiorum partitivirus S	NC_013014, NC_013015	5180	Sclerotinia sclerotiorum		Fungi
persistent	1661062	Sclerotinia sclerotiorum fusarivirus 1	NC_027208	5180	Sclerotinia sclerotiorum		Fungi
persistent	1685502	Pseudogymnoascus destructans partitivirus-pa	NC_030479, NC_030480	655981	Pseudogymnoascus destructans		Fungi
persistent	1766766	Colletotrichum gloeosporioides chrysovirus 1	NC_043666, NC_043667, NC_043668	474922	Colletotrichum gloeosporioides		Fungi
persistent	2304034	Colletotrichum fructicola chrysovirus 1	NC_040485, NC_040486, NC_040487, NC_040488, NC_040489, NC_040490, NC_040491	690256	Colletotrichum fructicola		Fungi
persistent	1565088	Colletotrichum higginsianum non-segmented dsRNA virus 1	NC_028242	759273	Colletotrichum higginsianum IMI 349063	27253323	Fungi
persistent	1917080	Sodiomyces alkalinus fusarivirus 1	NC_040529	1302862	Sodiomyces alkalinus		Fungi
persistent	759389	Verticillium dahliae chrysovirus 1	NC_038782, NC_038783, NC_038784, NC_038785	27337	Verticillium dahliae		Fungi
persistent	37961	Atkinsonella hypoxylon virus	NC_003470, NC_003471	47741	Atkinsonella hypoxylon	7782774	Fungi
persistent	382962	Epichloe festucae virus 1	NC_038930	35717	Epichloe festucae		Fungi
persistent	2305465	Ustilaginoidea virens nonsegmented virus 2	NC_040846	1159556	Ustilaginoidea virens	30128609	Fungi
persistent	1670975	Ustilaginoidea virens unassigned RNA virus HNND-1	NC_027427	1159556	Ustilaginoidea virens		Fungi
persistent	1312445	Ustilaginoidea virens RNA virus 1	NC_020997	1159556	Ustilaginoidea virens		Fungi
persistent	1460374	Ustilaginoidea virens RNA virus 3	NC_023547	1159556	Ustilaginoidea virens		Fungi
persistent	1756615	Ustilaginoidea virens RNA virus 5	NC_028477	1159556	Ustilaginoidea virens		Fungi
persistent	1561171	Ustilaginoidea virens RNA virus L	NC_025366	1159556	Ustilaginoidea virens		Fungi
persistent	1561172	Ustilaginoidea virens RNA virus M	NC_025367	1159556	Ustilaginoidea virens		Fungi
persistent	1415665	Ustilaginoidea virens partitivirus 2	NC_021873, NC_021874	1159556	Ustilaginoidea virens		Fungi
persistent	1054649	Beauveria bassiana RNA virus 1	NC_027428	176275	Beauveria bassiana	25577168	Fungi
persistent	1685109	Beauveria bassiana victorivirus 1	NC_038929	176275	Beauveria bassiana	23001673	Fungi
persistent	1485922	Beauveria bassiana victorivirus NZL/1980	NC_024151	176275	Beauveria bassiana		Fungi
persistent	1930960	Isaria javanica chrysovirus 1	NC_033277, NC_033278, NC_033317, NC_033318	43265	Cordyceps javanica		Fungi
persistent	2305250	Trichoderma asperellum dsRNA virus 1	NC_040828	101201	Trichoderma asperellum	30128610	Fungi
persistent	1934322	Trichoderma atroviride mycovirus	NC_033415	63577	Trichoderma atroviride	28050736	Fungi
persistent	2315392	Trichoderma harzianum bipartite mycovirus 1	NC_040768, NC_040769	5544	Trichoderma harzianum	30746561	Fungi
persistent	1562380	Fusarium coeruleum mitovirus 1	NC_026622	57164	Fusarium coeruleum		Fungi
persistent	2546034	Fusarium redolens polymycovirus 1	NC_055274, NC_055275, NC_055276, NC_055277, NC_055278, NC_055279, NC_055280, NC_055281	48865	Fusarium redolens		Fungi
persistent	1562379	Fusarium globosum mitovirus 1	NC_026621	78864	Fusarium globosum		Fungi
persistent	434898	Fusarium oxysporum chrysovirus 1	NC_043218, NC_043219, NC_043220	61369	Fusarium oxysporum f. sp. melonis		Fungi
persistent	1679238	Fusarium oxysporum f. sp. dianthi mycovirus 1	NC_027563, NC_027564, NC_027565, NC_027566	42551	Fusarium oxysporum f. sp. dianthi		Fungi
persistent	194397	Fusarium graminearum dsRNA mycovirus-1	NC_006937	5518	Fusarium graminearum	17646704	Fungi
persistent	687917	Fusarium graminearum dsRNA mycovirus-3	NC_013469	5518	Fusarium graminearum		Fungi
persistent	1848042	Fusarium poae dsRNA virus 2	NC_030201	36050	Fusarium poae		Fungi
persistent	1848169	Fusarium poae dsRNA virus 3	NC_030202	36050	Fusarium poae		Fungi
persistent	1849542	Fusarium poae mycovirus 1	NC_030869	36050	Fusarium poae	27550368	Fungi
persistent	1849543	Fusarium poae mycovirus 2	NC_030870	36050	Fusarium poae	27550368	Fungi
persistent	254946	Fusarium graminearum dsRNA mycovirus 2	NC_055219, NC_055220, NC_055221, NC_055222,	5518	Fusarium graminearum		Fungi
persistent	2268743	Fusarium asiaticum victorivirus 1	NC_040653	282267	Fusarium asiaticum		Fungi
persistent	1849535	Fusarium poae victorivirus 1	NC_030867	36050	Fusarium poae		Fungi
persistent	1872710	Fusarium graminearum deltaflexivirus 1	NC_030654	5518	Fusarium graminearum		Fungi
persistent	1809243	Fusarium graminearum mycotymovirus 1	NC_040784	5518	Fusarium graminearum		Fungi
persistent	1849531	Fusarium poae narnavirus 1	NC_030865	36050	Fusarium poae		Fungi
persistent	1849532	Fusarium poae narnavirus 2	NC_030866	36050	Fusarium poae		Fungi
persistent	1848150	Fusarium poae mitovirus 1	NC_030861	36050	Fusarium poae		Fungi
persistent	1848151	Fusarium poae mitovirus 2	NC_030862	36050	Fusarium poae		Fungi
persistent	1848152	Fusarium poae mitovirus 3	NC_030863	36050	Fusarium poae		Fungi
persistent	1848153	Fusarium poae mitovirus 4	NC_030864	36050	Fusarium poae		Fungi
persistent	687918	Fusarium graminearum dsRNA mycovirus 4	NC_013470, NC_013471	5518	Fusarium graminearum		Fungi
persistent	1284208	Fusarium graminearum hypovirus 1	NC_023680	5518	Fusarium graminearum		Fungi
persistent	1640379	Fusarium graminearum hypovirus 2	NC_026813	5518	Fusarium graminearum		Fungi
persistent	1926644	Fusarium langsethiae hypovirus 1	NC_032212	179993	Fusarium langsethiae		Fungi
persistent	75747	Fusarium poae virus 1	NC_003883, NC_003884	36050	Fusarium poae	10334037	Fungi
persistent	1849534	Fusarium poae virus 1-240374	NC_030877, NC_030882	36050	Fusarium poae		Fungi
persistent	1849533	Fusarium poae partitivirus 2	NC_030878, NC_030879	36050	Fusarium poae		Fungi
persistent	1849537	Fusarium poae fusarivirus 1	NC_030868	36050	Fusarium poae		Fungi
persistent	1849544	Fusarium poae negative-stranded virus 1	NC_030871	36050	Fusarium poae		Fungi
persistent	1849545	Fusarium poae negative-stranded virus 2	NC_030872	36050	Fusarium poae		Fungi
persistent	1511847	Fusarium solani virus 1	NC_003885, NC_003886	169388	Fusarium solani		Fungi
persistent	939923	Tolypocladium cylindrosporum virus 1	NC_014823	38005	Tolypocladium cylindrosporum		Fungi
persistent	267285	Chalara elegans RNA Virus 1	NC_005883	124036	Berkeleyomyces basicola		Fungi
persistent	274584	Thielaviopsis basicola mitovirus	NC_012585	124036	Berkeleyomyces basicola		Fungi
persistent	674982	Ceratocystis resinifera virus 1	NC_010754, NC_010755	1580851	Endoconidiophora resinifera		Fungi
persistent	235434	Ceratocystis polonica partitivirus	NC_010705, NC_010706	1580850	Endoconidiophora polonica		Fungi
persistent	40268	Cryphonectria hypovirus 2-NB58	L29010	5115	Cryphonectria		Fungi
persistent	1329781	Cryphonectria parasitica bipartite mycovirus 1	NC_021222, NC_021223	5116	Cryphonectria parasitica		Fungi

persistent		399394	Cryphonectria nitschkei chrysovirus 1	NC_038778, NC_038779, NC_038780, NC_038781	187221	Cryphonectria nitschkei		Fungi
persistent		311228	Mycoreovirus 1	NC_010743, NC_010744, NC_010745, NC_010746, NC_010747, NC_010748, NC_010749, NC_010750, NC_010751, NC_010752, NC_010753	5116	Cryphonectria parasitica		Fungi
persistent		404238	Cryphonectria parasitica mycoreovirus 2 (C18)	NC_038657	5116	Cryphonectria parasitica		Fungi
persistent		186769	Cryphonectria parasitica mitovirus 1-NB631	NC_004046	5116	Cryphonectria parasitica		Fungi
persistent		40281	Cryphonectria hypovirus 1	NC_001492	5116	Cryphonectria parasitica		Fungi
persistent		40282	Cryphonectria hypovirus 2	NC_003534	5116	Cryphonectria parasitica	8184535	Fungi
persistent		106962	Cryphonectria hypovirus 3	NC_000960	5116	Cryphonectria parasitica	10603318	Fungi
persistent		245101	Cryphonectria hypovirus 4	NC_006431	5116	Cryphonectria parasitica	15914232	Fungi
persistent		1779340	Phomopsis longicolla RNA virus 1	NC_033729	54899	Diaporthe longicolla		Fungi
persistent		1523858	Phomopsis longicolla hypovirus	NC_024685	54899	Diaporthe longicolla		Fungi
persistent		111470	Diaporthe ambigua RNA virus 1	NC_001278	73123	Diaporthe ambigua	11086142	Fungi
persistent		148880	Discula destructiva virus 1	NC_002797, NC_002800	148313	Discula destructiva		Fungi
persistent		160484	Discula destructiva virus 2	NC_003710, NC_003711	148313	Discula destructiva		Fungi
persistent		2587540	Coniothyrium diplodiella chrysovirus 1	NC_055624, NC_055625, NC_055626, NC_055627	332405	Coniella diplodiella		Fungi
persistent		1129873	Valsa ceratosperma hypovirus 1	NC_017099	1928361	Cytospora ceratosperma		Fungi
persistent		1580605	Phomopsis vexans RNA virus	NC_026135	222583	Phomopsis vexans		Fungi
persistent		764348	Magnaporthe oryzae chrysovirus 1	NC_014462, NC_014463, NC_014464, NC_014465, NC_023039, NC_023040, NC_023041, NC_023042,	318829	Pyricularia oryzae		Fungi
persistent		271257	Magnaporthe oryzae virus 1	NC_006367	318829	Pyricularia oryzae	17713838	Fungi
persistent		441999	Magnaporthe oryzae virus 2	NC_010246	318829	Pyricularia oryzae		Fungi
persistent		1661396	Magnaporthe oryzae virus 3	NC_027209	318829	Pyricularia oryzae		Fungi
persistent		1858671	Magnaporthe oryzae ourmia-like virus	NC_043683	318829	Pyricularia oryzae		Fungi
persistent		2509266	Magnaporthe oryzae polymycovirus 1	NC_055282, NC_055283, NC_055284, NC_055285	318829	Pyricularia oryzae		Fungi
persistent		198597	Ophiostoma mitovirus 3a	NC_004049	42373	Ophiostoma novo-ulmi		Fungi
persistent		88387	Ophiostoma mitovirus 4	NC_004052	42373	Ophiostoma novo-ulmi	10329574	Fungi
persistent		88388	Ophiostoma mitovirus 5	NC_004053	42373	Ophiostoma novo-ulmi	10329574	Fungi
persistent		88389	Ophiostoma mitovirus 6	NC_004054	42373	Ophiostoma novo-ulmi	10329574	Fungi
persistent		347482	Ophiostoma partitivirus 1	NC_038917, NC_038918	61193	Ophiostoma himal-ulmi		Fungi
persistent		1765736	Nigrospora oryzae victorivirus 1	NC_030224	335854	Nigrospora oryzae		Fungi
persistent		1913649	Nigrospora oryzae fusarivirus 1	NC_031960	335854	Nigrospora oryzae		Fungi
persistent		2086640	Entoleuca phenui-like virus 1	NC_055435, NC_055436	2021366	Entoleuca sp.		Fungi
persistent		1148496	Yado-kari virus 1	NC_040360	77044	Rosellinia necatrix	27571749	Fungi
persistent		2094187	Yado-nushi virus 1-A	NC_040357	77044	Rosellinia necatrix	27571749	Fungi
persistent		658904	Rosellinia necatrix megabirnavirus 1/W779	NC_013462, NC_013463	77044	Rosellinia necatrix		Fungi
persistent		1676267	Rosellinia necatrix megabirnavirus 2-W8	NC_029125, NC_029126	77044	Rosellinia necatrix		Fungi
persistent		1000373	Rosellinia necatrix quadrivirus 1	NC_016757, NC_016758, NC_016759, NC_016760	77044	Rosellinia necatrix		Fungi
persistent		1148491	Rosellinia necatrix victorivirus 1	NC_021565	77044	Rosellinia necatrix		Fungi
persistent		311229	Mycoreovirus 3	NC_007524, NC_007525, NC_007526, NC_007527, NC_007528, NC_007529, NC_007530, NC_007531, NC_007532, NC_007533, NC_007534, NC_007535, NC_007536	77044	Rosellinia necatrix		Fungi
persistent		1708712	Rosellinia necatrix endornavirus 1	NC_030938	77044	Rosellinia necatrix		Fungi
persistent		2032989	Rosellinia necatrix hypovirus 1	NC_036590	77044	Rosellinia necatrix		Fungi
persistent		859651	Rosellinia necatrix partitivirus 2	NC_020234, NC_020235	77044	Rosellinia necatrix	23236074	Fungi
persistent		235994	Rosellinia necatrix partitivirus 1-W8	NC_007537, NC_007538	77044	Rosellinia necatrix		Fungi
persistent		1573459	Rosellinia necatrix partitivirus 6	NC_028250, NC_028251	77044	Rosellinia necatrix		Fungi
persistent		2025333	Rosellinia necatrix partitivirus 8	NC_036600, NC_036603	77044	Rosellinia necatrix		Fungi
persistent		1476202	Rosellinia necatrix fusarivirus 1	NC_024485	77044	Rosellinia necatrix		Fungi
persistent		927810	Tuber aestivum virus 1	NC_038698	59557	Tuber aestivum		Fungi
persistent		941765	Tuber aestivum betaendornavirus	NC_014904	59557	Tuber aestivum		Fungi
persistent		1037523	Tuber aestivum mitovirus	NC_015629	59557	Tuber aestivum		Fungi
persistent		1300323	Scheffersomyces segobiensis virus L	NC_038697	45512	Scheffersomyces segobiensis		Fungi
persistent		12450	Saccharomyces cerevisiae killer virus M1	NC_001782	4932	Saccharomyces cerevisiae		Fungi
persistent		11008	Saccharomyces cerevisiae virus L-A	NC_003745	4932	Saccharomyces cerevisiae		Fungi
persistent		42478	Saccharomyces cerevisiae virus L-BC (La)	NC_001641	4932	Saccharomyces cerevisiae		Fungi
persistent		1921825	Saccharomyces kudriavzevii virus L-A1	NC_032106	114524	Saccharomyces kudriavzevii		Fungi
persistent		186772	Saccharomyces 20S RNA narnavirus	NC_004051	4932	Saccharomyces cerevisiae		Fungi
persistent		198599	Saccharomyces 23S RNA narnavirus	NC_004050	4932	Saccharomyces cerevisiae		Fungi
persistent		114871	Zygosaccharomyces bailii virus Z	NC_003874	4932	Saccharomyces cerevisiae		Fungi
persistent		1945738	Agaricus bisporus endornavirus 1	NC_055468	5341	Agaricus bisporus	28550284	Fungi
persistent		32625	Mushroom bacilliform virus	NC_001633	5341	Agaricus bisporus		Fungi
persistent		2547431	Lentinula edodes negative-strand RNA virus 2	NC_055449, NC_055450	5353	Lentinula edodes		Fungi
persistent		568090	Flammulina velutipes browning virus	NC_038825, NC_038826	38945	Flammulina velutipes		Fungi
persistent		674983	Pleurotus ostreatus virus 1	NC_006960, NC_006961	5322	Pleurotus ostreatus		Fungi
persistent		218667	Oyster mushroom spherical virus	NC_004560	5322	Pleurotus ostreatus		Fungi
persistent	A novel mycovirus from Clitocybe odora	1162083	Clitocybe odora virus	NC_017003	181985	Clitocybe odora		Fungi
persistent		1408133	Rhizoctonia cerealis alphaendornavirus 1	NC_022619	76351	Ceratobasidium cereale		Fungi
persistent		1908806	Ceratobasidium endornavirus A	NC_031462	1768090	Ceratobasidium sp.		Fungi
persistent		1908807	Ceratobasidium endornavirus B	NC_031463	1768090	Ceratobasidium sp.		Fungi
persistent		1908813	Ceratobasidium endornavirus C	NC_031461	1768090	Ceratobasidium sp.		Fungi

persistent	1908808	Ceratobasidium endornavirus D	NC_031449	1768090	Ceratobasidium sp.		Fungi
persistent	1908811	Ceratobasidium endornavirus G	NC_031464	1768090	Ceratobasidium sp.		Fungi
persistent	1837089	Rhizoctonia oryzae-sativae mitovirus 1	NC_029991	63189	Ceratorhiza oryzae-sativae		Fungi
persistent	1807787	Rhizoctonia solani endornavirus 2	NC_055462	456999	Rhizoctonia solani		Fungi
persistent	2162642	Rhizoctonia solani endornavirus 1	NC_040609	456999	Rhizoctonia solani		Fungi
persistent	1871631	Rhizoctonia solani flexivirus 1	NC_030655	456999	Rhizoctonia solani		Fungi
persistent	2421278	Rhizoctonia mitovirus 1	NC_040563	456999	Rhizoctonia solani		Fungi
persistent	1712387	Binucleate Rhizoctonia mitovirus K1	NC_027921	199337	Rhizoctonia sp. AG-K	34773510	Fungi
persistent	1708387	Rhizoctonia solani ourmia-like virus 1	NC_055143	456999	Rhizoctonia solani		Fungi
persistent	1825688	Rhizoctonia solani dsRNA virus 3	NC_032149, NC_032150	456999	Rhizoctonia solani		Fungi
persistent	46618	Rhizoctonia solani virus 717	NC_003801	456999	Rhizoctonia solani	10644855	Fungi
persistent	2045504	Rhizoctonia solani dsRNA virus 4	NC_040397, NC_040398	456999	Rhizoctonia solani		Fungi
persistent	1411681	Rhizoctonia solani dsRNA virus 2	NC_023684, NC_023685	983506	Rhizoctonia solani AG-1 IA	24889241	Fungi
persistent	349681	Phlebiopsis gigantea mycovirus dsRNA 1	NC_013999	82310	Phlebiopsis gigantea		Fungi
persistent	942041	Heterobasidion partitivirus 1	NC_038827, NC_038828	207833	Heterobasidion abietinum		Fungi
persistent	1469905	Heterobasidion partitivirus 12	NC_038829, NC_038830	13563	Heterobasidion annosum		Fungi
persistent	1469906	Heterobasidion partitivirus 13	NC_038831, NC_038832	13563	Heterobasidion annosum		Fungi
persistent	631431	Heterobasidion partitivirus 3	NC_038835, NC_038836	745715	Heterobasidion ecrustosum		Fungi
persistent	1469908	Heterobasidion partitivirus 15	NC_038833, NC_038834	207832	Heterobasidion parviporum		Fungi
persistent	1249677	Heterobasidion partitivirus 8	NC_038841, NC_038842	984962	Heterobasidion irregulare		Fungi
persistent	872291	Heterobasidion partitivirus 2	NC_038839, NC_038840	207832	Heterobasidion parviporum		Fungi
persistent	1387301	Heterobasidion partitivirus 7	NC_035485, NC_035486	207832	Heterobasidion parviporum		Fungi
persistent	186815	Heterobasidion annosum P-type partitivirus	NC_043393	13563	Heterobasidion annosum		Fungi
persistent	1770613	Thelephora terrestris virus 1	NC_028921	56493	Thelephora terrestris		Fungi
persistent	1895000	Sclerotium hydrophilum virus 1	NC_030888, NC_030891	5566	Sclerotium hydrophilum	27473231	Fungi
persistent	1167690	Xanthophyllomyces dendrorhous virus L1A	NC_020903	264483	Phaffia rhodozyma		Fungi
persistent	1167691	Xanthophyllomyces dendrorhous virus L1B	NC_038699	264483	Phaffia rhodozyma		Fungi
persistent	196690	Helicobasidium mompa totivirus 1-17	NC_005074	54444	Helicobasidium mompa	12876450	Fungi
persistent	675833	Helicobasidium mompa alphaendornavirus 1	NC_013447	54444	Helicobasidium mompa		Fungi
persistent	91913	Helicobasidium mompa dsRNA mycovirus	NC_043392	54444	Helicobasidium mompa		Fungi
persistent	1816484	Cronartium ribicola mitovirus 1	NC_030393	27354	Cronartium ribicola	27196406	Fungi
persistent	1816485	Cronartium ribicola mitovirus 2	NC_030395	27354	Cronartium ribicola	27196406	Fungi
persistent	1816486	Cronartium ribicola mitovirus 3	NC_030396	27354	Cronartium ribicola	27196406	Fungi
persistent	1816487	Cronartium ribicola mitovirus 4	NC_030397	27354	Cronartium ribicola	27196406	Fungi
persistent	1816488	Cronartium ribicola mitovirus 5	NC_030399	27354	Cronartium ribicola	27196406	Fungi
persistent	28882	Ustilago maydis virus H1	NC_003823	5270	Ustilago maydis		Fungi
persistent	1872708	Antonospora locustae virus 1	NC_035189	278021	Antonospora locustae	28267607	Fungi
persistent	2082658	Gigaspora margarita giardia-like virus 1	NC_040632	4874	Gigaspora margarita		Fungi
persistent	2082665	Gigaspora margarita mitovirus 1	NC_040702	4874	Gigaspora margarita		Fungi
persistent	2082666	Gigaspora margarita mitovirus 2	NC_040816	4874	Gigaspora margarita		Fungi
persistent	2082667	Gigaspora margarita mitovirus 3	NC_040559	4874	Gigaspora margarita		Fungi
persistent	2082668	Gigaspora margarita mitovirus 4	NC_040560	4874	Gigaspora margarita		Fungi
persistent	758865	Rhizophagus sp. RF1 medium virus	AB558119	1129544	Rhizophagus		Fungi
persistent	758866	Rhizophagus sp. RF1 mitovirus	NC_040656	1129544	Rhizophagus		Fungi
persistent	2320183	Rhizophagus diaphanum mitovirus 1	NC_040844	118614	Oehlia diaphana		Fungi
persistent	2320184	Rhizophagus diaphanum mitovirus 2	NC_040726	118614	Oehlia diaphana		Fungi
persistent	675060	Cryptosporidium parvum virus 1	NC_038843, NC_038844	5807	Cryptosporidium parvum		Alveolata
persistent	155414	Eimeria brunetti RNA virus 1	NC_002701	51314	Eimeria brunetti	19107323	Alveolata
persistent	1898175	Eimeria stiedai RNA virus 1	NC_040530	471275	Eimeria stiedai		Alveolata
persistent	1566868	Eimeria tenella RNA virus 1	NC_026140	5802	Eimeria tenella		Alveolata
persistent	1077832	Phytophthora infestans RNA virus 4	NC_029782	4787	Phytophthora infestans		Stramenopiles
persistent	2303160	Phytophthora infestans RNA virus 2	NC_040830	4787	Phytophthora infestans		Stramenopiles
persistent	1133557	Phytophthora infestans RNA virus 3	NC_040307	4787	Phytophthora infestans	23146209	Stramenopiles
persistent	640897	Phytophthora infestans RNA virus 1	NC_013220, NC_013221	4787	Phytophthora infestans		Stramenopiles
persistent	191289	Sclerophthora macrospora virus A	NC_005817, NC_005818, NC_005819	467176	Sclerophthora macrospora	12842628	Stramenopiles
persistent	75914	Sclerophthora macrospora virus B	NC_004714	467176	Sclerophthora macrospora	10562496	Stramenopiles
persistent	2083275	Pythium nunn virus 1	NC_040387, NC_040388	82942	Globisporangium nunn		Stramenopiles
persistent	2137353	Pythium polare RNA virus 1	NC_040608	1489738	Pythium polare		Stramenopiles
persistent	2137354	Pythium polare RNA virus 2	NC_040678	1489738	Pythium polare		Stramenopiles
